# Skin Aging and Carotenoids: A Systematic Review of Their Multifaceted Protective Mechanisms

**DOI:** 10.3390/nu17162596

**Published:** 2025-08-09

**Authors:** Cristina Stanescu, Iulia Chiscop, Daniela Mihalache, Florina Popa, Camelia Tamas, Gabriela Stoleriu

**Affiliations:** 1 Department of Morphological and Functional Sciences, Faculty of Medicine and Pharmacy, “Dunarea de Jos” University, 35 Alexandru Ioan Cuza Street, 800216 Galati, Romania; 2 Clinical Surgical Department, Faculty of Medicine and Pharmacy, “Dunărea de Jos” University, 47 Domneasca Street, 800008 Galati, Romania; 3 Department of Plastic Surgery, Faculty of Medicine, “Grigore T. Popa”, University of Medicine and Pharmacy, 16 Universitatii Street, 700115 Iasi, Romania; 4 Clinical Medical Department, Faculty of Medicine and Pharmacy, “Dunarea de Jos” University, 35 Alexandru Ioan Cuza Street, 800216 Galati, Romania

**Keywords:** carotenoids, protective roles, skin aging, lifestyle

## Abstract

Skin aging is a complex biological process influenced by intrinsic factorssuch as genetic predispositions and hormonal changes as well as extrinsic factors including ultraviolet radiation, environmental pollution, and lifestyle habits. This process culminates in a progressive decline in the structural and functional integrity of the skin. This review delves into the protective roles of carotenoids, highlighting their significant anti-oxidative, anti-inflammatory, and photoprotective properties. We included studies that investigated the effects of dietary or topical carotenoids on skin aging markers in human and animal models. Eligible studies were identified through searches of PubMed, Scopus, Web of Science, Embase, Google Scholar, and the Cochrane Library from January 2000 to March 2025. Risk of bias was assessed using the Cochrane RoB tool for randomized trials and animal studies. A total of 176 studies were included, and data were synthesized narratively due to heterogeneity in study designs and outcomes. The findings indicate that carotenoids mitigate oxidative stress-induced cellular damage by scavenging reactive oxygen species (ROS) and Reactive Nitrogen Species (RNS), attenuating chronic inflammation, and enhancing dermal matrix integrity via collagen biosynthesis and modulation of matrix metalloproteinases. Additionally, they support skin hydration and elasticity by indirectly regulating aquaporins and promoting hyaluronic acid synthesis. This review further explores emerging strategies that incorporate carotenoid supplementation in lifestyle medicine and preventive dermatology. By elucidating the cellular pathways through which carotenoids exert their effects and modulate mitochondrial function, this review highlights their translational potential in anti-aging skincare. Ongoing research is essential to comprehend the complex connections between carotenoids, skin physiology, and overall health. This understanding will ultimately facilitate the creation of personalized nutritional and dermocosmetic strategies.

## 1. Introduction

Carotenoids are naturally occurring pigments present in fruits and vegetables, recognized for their significant antioxidant and anti-inflammatory properties. Compounds such as β-carotene, lycopene, lutein, and astaxanthin have been shown to confer protective effects against oxidative damage, UV-induced photoaging, and inflammation in both topical and dietary applications. Although interest in the effects of carotenoids on skin aging is growing, the evidence remains dispersed across various study designs and delivery methods. While the benefits of carotenoids are well-documented, the precise mechanisms by which they impact aging pathways in human skin have not yet been fully elucidated. This review underscores the translational potential of carotenoids in anti-aging skincare by clarifying the cellular pathways through which they exert their effects and influence mitochondrial function.

### 1.1. Objective

This review aims to analyze the protective mechanisms of carotenoids against skin aging. It covers a range of topics, from the basic understanding of skin structure and the aging process to the detailed mechanisms through which carotenoids exert their protective effects, including anti-oxidative, anti-inflammatory, antitumoral, immunomodulatory, antilipidic, antiglucemic, and photoprotective activities, as well as their role in supporting collagen production and skin hydration. This review assessed the clinical efficacy of both dietary and topical carotenoids in promoting skin health and identified future research directions in this field. Although the benefits of carotenoids are well documented, the specific mechanisms through which they affect aging pathways in human skin have not yet been fully elucidated. This review aims to summarize recent research findings to enhance our understanding of these mechanisms.

### 1.2. Skin Functions

The human skin is a highly specialized and multifunctional organ composed of several distinct layers (epidermis, dermis, and hypodermis) and associated appendages, such as sweat glands, sebaceous glands, nails, and hair follicles. These structures collectively function as dynamic barriers against environmental insults, thermoregulatory systems, and sensorial interfaces that facilitate immune surveillance and neurosensory processing. The epidermal barrier continues to mature during early childhood, achieving adult-like functionality for transepidermal water loss (TEWL), lipid matrix organization, stratum corneum thickness, and corneocyte cohesion by approximately six years of age. In neonates and young children, increased lactic acid levels and reduced concentrations of total amino acids in the stratum corneum are indicative of heightened keratinocyte turnover and epidermal remodeling compared to adult skin [1,2,3]. Appendageal structures such as hair follicles and eccrine glands not only facilitate thermoregulation but also contribute to the cutaneous microbiome landscape and local immune responses [4].

Marked variations in hydration status, melanin distribution, skin thickness, and elasticity were observed across age groups, anatomical sites, and sex. Estrogens play a pivotal role in preserving dermal architecture, enhancing collagen synthesis, and maintaining vascular homeostasis [5]. The onset of menopause, accompanied by hypoestrogenism, exacerbates the intrinsic and extrinsic aging phenotypes, including dermal thinning, increased wrinkle depth, and impaired wound healing. Phytoestrogens have emerged as promising candidates to counteract estrogen deficiency-related cutaneous degeneration, particularly in postmenopausal women [6]. Gender-specific differences in sebaceous activity and hydration patterns have been documented, with men exhibiting higher sebum output and lower hydration levels after the age of 40 years than age-matched women. Aging also affects tactile perception, particularly in glabrous skin, where dryness and structural alterations reduce sensory acuity. In contrast, hairy skin retains some protective and functional advantages owing to its vascular and appendageal supply. This type of skin features a complex network of blood vessels that is crucial for maintaining skin homeostasis, promoting regeneration, and facilitating wound healing. Adequate vascularization enables effective blood flow and nutrient delivery, essential for the prolonged function and survival of skin tissues. The presence of a robust blood supply in hairy skin also supports the dynamic plasticity necessary for sensory and protective functions. In conclusion, the vascular and appendageal features of hairy skin contribute significantly to its protective functions, sensory feedback, and regenerative capabilities, underscoring its vital role in skin physiology [4,7].

Skin aging is a complex biological process that is influenced by the following factors:

- Intrinsic factors: genetics, hormonal changes, and metabolic processes
- Extrinsic factors: UV radiation, pollution, smoking, and poor nutrition

Understanding these nuances provides critical insights into the vulnerabilities and adaptive mechanisms of aging skin, laying the foundation for targeted preventive and therapeutic strategies.

### 1.3. Skin Aging Mechanisms and Molecular Pathways

Skin aging is a progressive biological phenomenon marked by structural disorganization and functional decline in cutaneous tissues, which include the following:

- Dermal thinning, loss of collagen and elastin, and decreased vascularity.
- Dryness, reduction in hyaluronic acid, and change in cell permeability.
- Pigmentation, xerosis, and elastosis.
- Increasing risk for non-melanomatous cutaneous carcinomas.
- Susceptibility to infections and irritations.
- Modifying the microbiome and immune response.
- Cellular senescence, stem cell depletion, mitochondrial dysfunction, cellular autophagy, telomere shortening, accumulation of glycation products, and increased oxidative stress.
- The consequence of these changes was the formation of wrinkles. This phenomenon is driven by a constellation of interrelated molecular and cellular mechanisms, including a decreased ability to neutralize free radicals, a reduction in anti-oxidant capacity, and chronic inflammation [8].

The understanding of these mechanisms is optimally framed within the context of theories such as the free radical theory of aging, the inflammaging hypothesis (a portmanteau of inflammation and aging, which refers to the chronic low-grade inflammation associated with aging, contributing to various age-related diseases), photoaging dynamics, and emerging metabolic frameworks. At the cellular level, chronological aging is associated with a decline in the number and functionality of keratinocytes, fibroblasts, and mast cells, resulting in reduced proliferative potential, impaired extracellular matrix (ECM) turnover, and diminished wound-healing capacity. Aged dermal fibroblasts exhibit attenuated migration, reduced contractility, increased senescence markers, and heightened vulnerability to oxidative insults [9].

#### 1.3.1. Oxidative Stress and Skin Aging

A central feature of skin aging is the accumulation of reactive oxygen species (ROS), which contribute to oxidative damage to proteins, lipids, and DNA. This results in extracellular matrix degradation, particularly collagen breakdown, through upregulation of matrix metalloproteinases (MMPs). Additionally, ROS promotes the secretion of pro-inflammatory cytokines, fueling chronic low-grade inflammation [10,11,12,13]. The reduction in growth differentiation factor 15 (GDF15) in dermal fibroblasts contributes to mitochondrial dysfunction, further exacerbating oxidative stress and compromising the bioenergetic balance. Key signaling networks implicated in skin aging include Nrf2 (nuclear factor erythroid 2–2-related factor 2), mechanistic target of rapamycin (mTOR), transforming growth factor-beta (TGF-β), and IGF-1 (insulin-like growth factor 1). These pathways orchestrate responses involving telomere attrition, autophagy dysregulation, pro-inflammatory gene expression, and altered ECM remodeling [14,15]. Glycation of dermal proteins, particularly long-lived collagen fibers, leads to the accumulation of advanced glycation end products (AGEs), contributing to tissue stiffness, diminished elasticity, and persistent oxidative stress via AGE–receptor interactions (RAGEs). The cycle of oxidative damage-induced DNA damage leads to the activation of p53, which subsequently initiates apoptosis or induces permanent cell cycle arrest (senescence), thereby contributing to further skin deterioration [16,17,18,19].

#### 1.3.2. Aquaporins Dysregulation in Skin Aging

Aquaporins (AQPs), a family of membrane water channels, are emerging as regulators of skin aging. AQP dysregulation influences epidermal hydration, keratinocyte proliferation, immune cell migration, and epithelial–mesenchymal interactions. Impaired AQP function is correlated with mitochondrial dysfunction, stem cell depletion, macrophage dysregulation, and heightened inflammation [20,21,22,23,24].

Aquaporins (AQPs) are integral membrane proteins that facilitate the transport of water and certain small solutes such as glycerol across cellular membranes and play crucial roles in maintaining skin hydration and epidermal homeostasis. Dysregulation of AQP expression and function can significantly affect skin physiology and is associated with various dermatological conditions. AQP3, one of the predominant aquaglyceroporins in the epidermis, is pivotal for maintaining skin hydration by facilitating the transport of both water and glycerol [20]. Studies have shown that deletion of AQP3 in mice results in impaired skin hydration and elasticity, with a marked decrease in glycerol and water content within the stratum corneum (SC), leading to defective skin barrier recovery [21]. This deficiency in AQP3 expression leads to an increased rate of transepidermal water loss, highlighting its importance in retaining moisture within skin layers. Furthermore, research indicates that pharmacological interventions aimed at modulating AQP3 expression can improve skin barrier function [22]. For instance, the topical application of glyceryl glucoside was found to upregulate AQP3 mRNA and protein levels, thereby enhancing skin hydration and reducing water loss through improved barrier integrity. In addition to water transport, AQPs such as AQP3 also facilitate the movement of glycerol, an important component in maintaining the lipid balance of the epidermis, which is vital for skin hydration and elasticity. The impaired transport of glycerol due to AQP dysregulation contributes to the reduced hydration capacity and impaired barrier function observed under conditions of AQP deficiency [23,24]. Overall, the role of AQPs, especially AQP3, extends beyond water transport and encompasses the regulation of skin lipid metabolism and repair processes, which are critical for maintaining optimal skin hydration and overall skin health. Modulation of AQP activity presents a promising therapeutic strategy for treating various skin disorders characterized by dehydration and compromised barrier function. Hyaluronic acid (HA) depletion contributes to the loss of skin turgor, dehydration, and barrier impairment. Combining AQP modulators with HA-based formulations and lipophilic antioxidants such as vitamins A, C, and E may help restore hydration and cellular homeostasis [25,26].

#### 1.3.3. Melatonin in Skin Aging

Melatonin, a neurohormone primarily synthesized by the pineal gland, also exhibits extra-pineal production in skin cells, including keratinocytes, melanocytes, and fibroblasts, where it exerts potent anti-oxidant, anti-inflammatory, and cytoprotective effects. Cutaneous melatonin production contributes to local circadian regulation, DNA repair, and the neutralization of ultraviolet (UV)-induced oxidative stress. With advancing age, systemic and local melatonin synthesis declines, a process linked to increased susceptibility to photoaging, mitochondrial dysfunction, and loss of dermal resilience. This decline correlates with the formation of deep wrinkles, dehydration, and disrupted epidermal renewal cycle [27,28]. Melatonin neutralizes hydroxyl radicals, hydrogen peroxide, and singlet oxygen and indirectly enhances the activity of anti-oxidant enzymes such as glutathione peroxidase and superoxide dismutase, thereby strengthening cellular redox homeostasis. Beyond its anti-oxidant properties, melatonin significantly contributes to matrix remodeling, enhances type I collagen synthesis, and prevents UV-induced apoptosis, making it a promising ingredient for anti-aging dermatological formulations [29,30]. Given the multifactorial nature of skin aging, strategies that integrate melatonin supplementation with broad-spectrum anti-oxidant protection may offer synergistic effects to attenuate environmentally induced skin damage.

#### 1.3.4. Environmental Stressors in Skin Aging

Environmental stressors, including ambient particulate matter (PM), volatile organic compounds (VOCs), nitrogen oxides, ozone (O_3_), and prolonged light exposure (UV, blue, and infrared spectra) act synergistically to compromise skin integrity [31]. These agents activate cellular stress pathways, leading to protein oxidation, lipid peroxidation, and chronic inflammation, thereby accelerating extrinsic aging and promoting carcinogenesis. A central mediator in this process is the aryl hydrocarbon receptor (AhR), a ligand-activated transcription factor responsive to dioxins, polycyclic aromatic hydrocarbons (PAHs), and UV radiation. AhR activation disrupts epidermal differentiation, induces pigmentary changes, and enhances MMP expression, thereby accelerating collagen degradation and oxidative DNA damage. Inhibition of AhR signaling represents a novel strategy for mitigating environmental aging [32,33]. Exposure to environmental pollutants and tobacco smoke synergistically inhibits the TGF-β/Smad pathway, downregulates procollagen type I synthesis, and upregulates MMP-1 and inflammatory mediators, such as IL-6 and TNF-α. This results in matrix disorganization, dermal volume loss, and impaired wound repair. The cumulative oxidative burden of ROS derived from pollutants leads to mitochondrial decay, increased senescent cell load, and activation of DNA repair, and apoptotic cascades, particularly via p53 and MAPK signaling. These changes diminish the ability of the skin to regenerate and compromise its barrier and immune functions [34,35].

**Figure 1:**
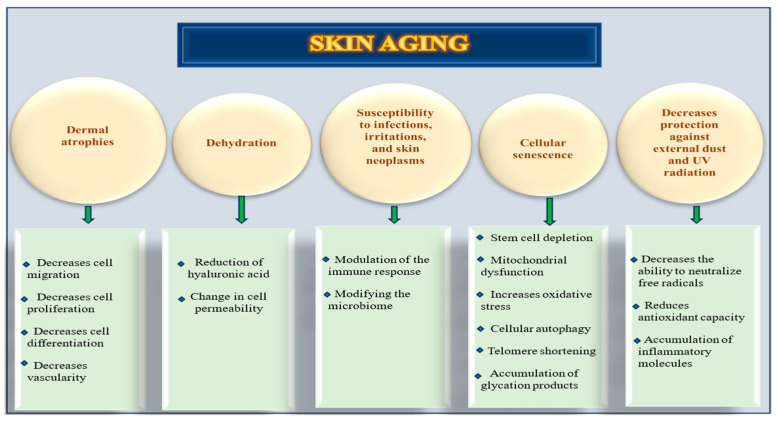
Structural and functional changes in skin with aging.

Skin aging is dictated by a complex and multilayered network of molecular events including redox imbalance, chronic inflammation, DNA instability, cellular senescence, and matrix degradation (Figure 1). Future therapeutic strategies must integrate these biological insights to develop targeted interventions aimed at decelerating cutaneous aging and preserving dermal integrity.

### 1.4. Protective Roles of Carotenoids

Carotenoids represent a class of naturally occurring pigments present in plants, algae, and certain bacteria. These organic compounds are characterized by a polyene chain of conjugated double bonds and can be broadly categorized into two primary groups:

1. Carotenes: These are hydrocarbon carotenoids composed solely of carbon and hydrogen atoms (e.g., β-carotene, lycopene).
2. Xanthophylls: These are oxygenated carotenoids that contain oxygen atoms in addition to carbon and hydrogen (e.g., lutein, zeaxanthin, astaxanthin).

Carotenoids exert a wide spectrum of biological actions that contribute to skin protection, rejuvenation, and long-term resilience through multifactorial mechanisms at the cellular and molecular levels. Their protective roles include both direct effects on skin cells and indirect systemic effects, which are mediated through metabolic and immunological pathways.

1. Photoprotection: Carotenoids, including β-carotene, lutein, and lycopene, absorb specific wavelengths of light and mitigate the phototoxic effects of ultraviolet radiation. This process helps limit DNA photodamage, oxidative stress, and inflammation related to photoaging. Their accumulation in skin tissue offers intrinsic protection against UV-induced erythema and pigmentary changes [36,37,38,39].
2. Anti-oxidant Activity: Carotenoids play a crucial role in maintaining cellular redox balance by scavenging reactive oxygen species (ROS), reducing lipid peroxidation, and enhancing the activity of endogenous anti-oxidant enzymes, such as superoxide dismutase and glutathione peroxidase. This function is especially important in the epidermal and dermal layers, which are often subjected to environmental stressors [11,40,41,42,43].
3. Anti-inflammatory Properties: By suppressing NF-κB and MAPK signaling, carotenoids reduce the expression of pro-inflammatory cytokines, such as TNF-α, IL-6, and IL-1β, while also inhibiting key enzymes, such as COX-2 and LOX. This action prevents chronic skin inflammation and the associated tissue degradation [44,45,46].
4. DNA Protection and Repair: Carotenoids contribute to the maintenance of genomic stability by minimizing oxidative DNA damage and preserving mitochondrial integrity. Some carotenoids may also affect DNA repair pathways, although this requires further investigation in human skin models [47,48,49,50,51].
5. Collagen and ECM Support: Carotenoids enhance fibroblast activity and promote collagen synthesis by activating TGF-β signaling while also inhibiting MMPs that break down structural proteins. This dual mechanism helps to preserve skin elasticity and dermal density, which are essential features of young skin [52,53,54,55,56].
6. Stimulation of Hyaluronic Acid (HA) Synthesis: Carotenoids indirectly boost HA production by enhancing fibroblast viability and diminishing inflammatory mediators, which are known to inhibit HA synthase. This process leads to improved skin hydration and turgor [57,58].
7. Neuroprotective and Mood-Stabilizing Effects: Recent evidence suggests that carotenoids may influence mood, cognition, and neuroinflammation, linking oxidative balance and psychological well-being to skin health through the gut–brain–skin axis [59,60,61,62,63,64].
8. Immune Modulation and Microbiome Support: Carotenoids promote immune homeostasis by influencing macrophage polarization, T cell function, and gut microbial diversity, which may indirectly enhance skin barrier integrity and immune tolerance [65,66,67,68].
9. Nutrient Bioavailability and Metabolic Interactions: Carotenoids enhance the overall anti-oxidant capacity and demonstrate synergistic effects when combined with other micronutrients such as vitamins C and E, selenium, and polyphenols. These interactions amplify protective effects at multiple cellular checkpoints [69,70].

Carotenoids act through an integrated network of photoprotective, anti-oxidative, anti-inflammatory, structural, and immunoregulatory mechanisms that collectively contribute to slowing skin aging, enhancing skin regeneration, and maintaining cutaneous homeostasis.

**Figure 2:**
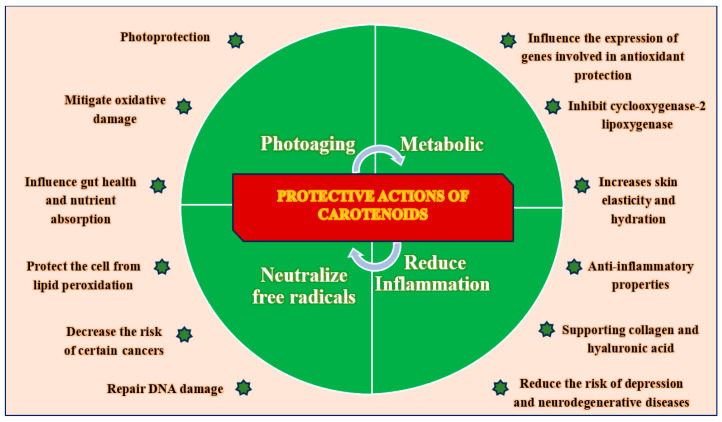
Protective roles of carotenoids.

These roles are summarized visually in the updated Figure 2: “Protective Roles of Carotenoids.”

### 1.5. Food Sources of Carotenoids

**Figure 3:**
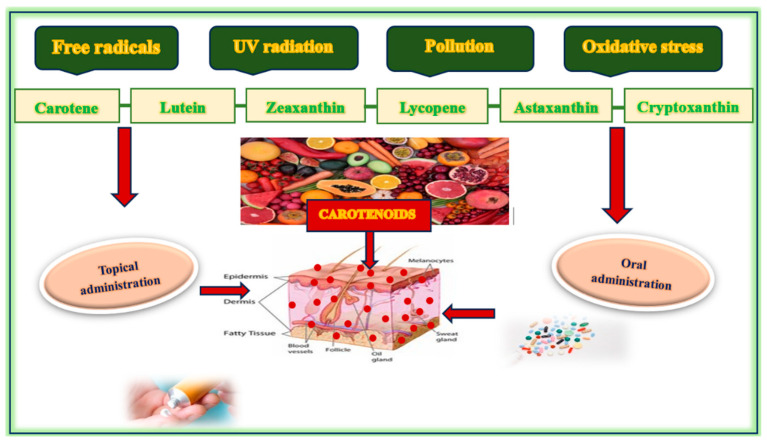
The type of carotenoids involved in the defense process in the skin.

Considering the significant protective benefits of carotenoids for skin health, it is essential to identify dietary sources that can seamlessly integrate these crucial nutrients into daily routines, highlighting their practical importance in skincare. Carotenoids are exclusively derived from dietary sources, and plant-based foods, fungi, algae, and certain species of microorganisms constitute the primary reservoir. These compounds are synthesized by photosynthetic organisms, and their concentrations in human tissues depend on dietary intake, intestinal absorption, and systemic distribution. The most bioavailable and skin-relevant carotenoids include β-carotene, α-carotene, lycopene, lutein, zeaxanthin, astaxanthin, and cryptoxanthin [71] (Figure 3).

Carrots, pumpkins, and sweet potatoes are rich in α- and β-carotene, which are provitamin A carotenoids capable of being converted into retinol. Tomatoes and processed tomato products (e.g., tomato paste and sauce) are the primary sources of lycopene, a carotenoid known for its strong singlet oxygen quenching ability. Dark leafy greens, such as spinach, kale, and Swiss chard, are rich in lutein and zeaxanthin, which are two xanthophyll carotenoids that are particularly effective in protecting against photo-oxidative stress and blue light-induced damage. Red and yellow peppers, citrus fruits, and orange juice contain appreciable levels of β-cryptoxanthin, another provitamin A carotenoid with anti-oxidant properties. Papaya, mangoes, and apricots are notable sources of carotenes, particularly in tropical and subtropical diets. Carotenoids derived from algae have garnered significant commercial interest due to their bioactive properties, which render them valuable for applications in the food, feed, nutraceutical, pharmaceutical, and cosmeceutical industries. Microalgae, such as *Spirulina platensis*, *Haematococcus pluvialis*, *Dunaliella salina*, *Chlorella vulgaris*, *Chlorella pyrenoidosa*, and *Chlorella sorokiniana*, are particularly known for their high carotenoid content. These carotenoids include β-carotene, astaxanthin, canthaxanthin, and lutein, among others. The production of carotenoids from algae is environmentally friendly, cost-effective, and yields higher outputs compared to other sources. While the production of carotenoids from algae presents distinct advantages, continuous research is essential to optimize production methods and enhance the economic feasibility of algal biotechnology. The current trends and prospects in this field are centered on overcoming the technological and economic barriers to fully harness algae as a sustainable source of carotenoids. Other sources with a high carotenoid content include marine organisms, such as salmon, krill, and shrimp, and offer a highly potent anti-oxidant effect relevant to skin aging [72,73,74].

The bioavailability of carotenoids is influenced by several factors, including the food matrix, the presence of dietary fat, food processing methods, and individual digestive efficiency. For instance, carotenoid micellarization, a key step in intestinal absorption, is significantly enhanced when carotenoids are consumed with unsaturated dietary fats, such as olive oil, sunflower oil, and soybean oil. These fats promote the formation of mixed micelles, thereby facilitating carotenoid uptake by enterocytes. Papaya exhibits superior carotenoid micellarization efficiency compared to other fruits and vegetables, particularly lutein, β-carotene, and lycopene. Moreover, thermal processing methods, such as steaming and light cooking, often enhance carotenoid bioavailability by breaking down plant cell walls and protein–carotenoid complexes. However, excessive heat can lead to oxidative degradation [75,76,77].

Seasonal variation also plays a role, as the consumption of fresh carotenoid-rich produce peaks during spring and summer, coinciding with increased cutaneous exposure to UV radiation, which may upregulate endogenous mechanisms of carotenoid deposition in the skin. Genetic polymorphisms affecting enzymes, such as β-carotene monooxygenase 1 (BCMO1), influence individual variability in carotenoid conversion efficiency and systemic levels, which may further affect dermatological outcomes and anti-oxidant status. A diet rich in diverse carotenoids, enhanced by unsaturated fats and minimally processed plant foods, plays a crucial role in boosting the anti-oxidant capacity of the skin, thereby aiding in photoprotection, hydration, and collagen preservation. Targeted nutritional interventions may therefore serve as adjunctive strategies in anti-aging skincare and preventive dermatology [78,79,80,81]. Recent advancements in analytical techniques have revealed novel carotenoids and their metabolites, necessitating an updated review of their potential roles in skin health and aging.

### 1.6. Carotenoids in Skincare Products

While dietary intake remains as the primary means for systemic carotenoid accumulation, there is an increasing interest in topical application of carotenoids to deliver targeted anti-oxidant, anti-inflammatory, and photoprotective effects directly to the skin. Carotenoids are increasingly being incorporated into cosmeceutical formulations and products that lie at the intersection of cosmetics and pharmaceuticals, owing to their ability to modulate key biological pathways involved in aging, inflammation, and oxidative stress. Clinical studies have demonstrated that carotenoids can effectively enhance the skin’s resilience against UV-induced damage. Erythema, commonly known as sunburn, resulting from UVB exposure, and UVA-induced pigmentation are the primary clinical indicators employed in these studies. The findings from this research indicate an increase in skin carotenoid levels, alongside improvements in the minimal erythemal dose (MED) and minimal persistent pigmentation dose (MPPD), which are measures of skin resistance to UV radiation. Incorporating carotenoids into daily dietary routines offers a natural approach to enhancing skin resilience and reducing the risk of photodamage. Topical formulations enriched with carotenoids, such as astaxanthin, lycopene, β-carotene, and lutein, have demonstrated efficacy [38,46,69,82].

− Enhancing the skin’s anti-oxidant defense, particularly in the stratum corneum and viable epidermis, where UV- and pollution-induced oxidative stress are the most concentrated [40,41,42,43].
− Improving skin elasticity and hydration through membrane stabilization and interaction with structural lipids [20,21,22,23,24].
  Matrix metalloproteinases (MMPs) and pro-inflammatory cytokines are downregulated, thereby preserving dermal collagen and inhibiting extracellular matrix degradation [44,45,53,54,56]. When applied topically, carotenoids penetrate the stratum corneum and accumulate within skin lipids, where they exert localized protection against photo-oxidative damage. Carotenoid concentrations in the skin vary depending on diet, sun exposure, and metabolism. A diet rich in fruits and vegetables significantly raises carotenoid levels, while stress factors, including illness and UV radiation, can cause a rapid decrease. Bioavailability is increased in the presence of dietary fats and can be influenced by the individual’s physiological state. Additionally, higher cardiovascular fitness and lower body fat can increase skin carotenoid levels, enhancing skin yellowness and contributing to a healthier appearance [83,84]. β-Carotene, predominantly located in the stratum corneum and dermis, functions as a precursor to retinoic acid. The concentration of this compound in skin tissue varies significantly, ranging from 0.2 to 1.0, with elevated levels observed in individuals who consume diets rich in carotenoids. Lycopene is predominantly localized in the deeper layers of the epidermis and demonstrates a significant capacity for quenching singlet oxygen. Its concentration typically ranges from 0.1 to 0.8 µg/g, which is often lower than that of β-carotene, yet it exhibits comparable anti-oxidant efficacy. Lutein and zeaxanthin, although present in lower concentrations, play a crucial role in protecting against blue light exposure and oxidative damage. Astaxanthin, although less prevalent, exhibits exceptional anti-oxidant activity and has been researched for its anti-aging properties. It is found in nanomolar concentrations but offers remarkable anti-aging benefits due to its potent anti-oxidant capabilities. Carotenoids are integral to skin defense mechanisms, and their concentrations serve as biomarkers of nutritional status and oxidative resilience [46,69]. Their presence has been associated with reduced wrinkle depth, improved surface smoothness, and enhanced dermal resilience, particularly in aged or photodamaged skin. To improve skin bioavailability and penetration, carotenoids are frequently incorporated into advanced delivery systems, including the following:

- Nanoemulsions and liposomes enhance solubility and skin permeation.
- Solid lipid nanoparticles (SLNs) and nanostructured lipid carriers (NLCs) offer controlled release, photostability, and deep-tissue delivery.
- Hydrogel-based systems provide occlusion and prolong skin contact.

Topical carotenoids also interact synergistically with other skin-beneficial compounds such as vitamin C (ascorbic acid), vitamin E (tocopherol), coenzyme Q10, and flavonoids, amplifying their photoprotective and anti-oxidant effects. The long-term deposition of carotenoids in the epidermis, as observed through noninvasive Raman spectroscopy, provides a sustained reservoir of protection, which is particularly beneficial in the context of chronic UV exposure, urban pollution, and intrinsic aging. From a translational perspective, ongoing research has focused on identifying the optimal concentrations, combinations, and delivery vehicles to maximize the efficacy of carotenoid-based topical interventions. These formulations may soon become standard components of dermatological protocols aimed at preventing photoaging, reducing inflammation, and restoring skin homeostasis [46,69,83,84]. Carotenoids represent a promising class of bioactive cosmeceutical agents capable of bridging the gap between nutritional science and dermatologic therapy. Topical application complements oral intake in a dual-action strategy for comprehensive skin protection and rejuvenation.

Carotenoids, which are known for their anti-oxidative and anti-inflammatory properties, offer a promising solution to the intricate interplay of factors that contribute to skin aging. Ongoing research into their cutaneous distribution and functional roles may contribute to the development of innovative strategies for skin protection, anti-aging interventions, and dietary recommendations.

### 1.7. Mechanisms of Carotenoids

#### 1.7.1. Anti-Oxidant Protection of Carotenoids

Carotenoids are a class of lipophilic triterpenoid pigments that play a pivotal role in neutralizing reactive oxygen species (ROS), thereby attenuating oxidative stress, which is a central mechanism in the pathophysiology of skin aging. Their chemical structure, composed of conjugated double bonds, enables efficient quenching of singlet oxygen and peroxyl radicals, making them highly effective anti-oxidants in biological systems. As part of the skin’s endogenous anti-oxidant defense network, carotenoids protect against environmental aggressors, such as UV radiation, ozone, cigarette smoke, and heavy metals, which induce oxidative DNA damage, lipid peroxidation, and protein denaturation. By donating electrons to neutralize free radicals, carotenoids mitigate cellular oxidative injury and prevent DNA strand breaks, mitochondrial dysfunction, and mutagenesis [36,85,86].

Carotenoids, including beta-carotene and lycopene, vary in their effectiveness at neutralizing harmful oxygen-related molecules (ROS), which is influenced by their chemical structure and accumulation in the body. Beta-carotene and lycopene, for instance, are particularly potent in deactivating singlet oxygen, whereas astaxanthin exhibits superior activity against hydroxyl and peroxyl radicals. Carotenoids upregulate the expression of endogenous anti-oxidant enzymes, including superoxide dismutase (SOD), catalase (CAT), and glutathione peroxidase (GPx), by modulating transcription factors such as Nrf2 (nuclear factor erythroid 2-related factor 2). Through this pathway, carotenoids not only function as direct anti-oxidants but also indirectly enhance cellular resilience against oxidative insults. Importantly, anti-oxidants can exhibit dual behavior, acting as pro-oxidants under certain conditions, particularly at high concentrations or in the presence of metal ions. This underscores the importance of dose optimization and synergistic formulation. For example, ferulic acid demonstrates a strong scavenging capacity against hydroxyl radicals, whereas vitamin C neutralizes peroxynitrite, and anserine–carnosine complexes are effective against hypochlorous species. A combination of these agents tailored to target diverse ROS may offer broader anti-oxidant coverage [87,88,89,90].

The lipophilicity of carotenoids facilitates their incorporation into cell membranes, where they stabilize lipid bilayers and prevent lipid peroxidation at ROS generation sites. In contrast, hydrophilic anti-oxidants act within aqueous compartments, neutralizing ROS in the cytosol and extracellular matrix. Together, lipophilic and hydrophilic anti-oxidants form a complementary defense system that ensures holistic cellular protection. Exosomes and extracellular vesicles (EVs), particularly those derived from mesenchymal stem cells (MSCs), have emerged as mediators of anti-oxidant activity. Nanoscale vesicles carry mRNAs, microRNAs, and anti-oxidative proteins, helping restore redox balance and promote tissue repair. MSC-derived exosomes reduce MMP expression, stimulate collagen and elastin production, and enhance cell-to-cell signaling under oxidative stress [91,92,93,94].

Carotenoids serve as multi-targeted anti-oxidant agents with benefits beyond radical scavenging, including gene regulation, enzymatic activation, and membrane stabilization. Their incorporation into dietary and topical regimens holds significant potential for preserving dermal structure, delaying senescence, and enhancing skin resilience against environmental and endogenous aging triggers. Considering their anti-oxidant capabilities, carotenoids offer significant photoprotection, as discussed in the following section.

#### 1.7.2. Photoprotection

Photoprotection refers to the prevention or attenuation of skin damage caused by ultraviolet (UV) radiation, primarily ultraviolet A (UVA; 320–400 nm) and UVB (UVB; 280–320 nm), which are known to induce photoaging, immunosuppression, and carcinogenesis. Carotenoids have emerged as effective agents for intrinsic photoprotection and function via both physical and biochemical mechanisms. Certain carotenoids, notably β-carotene, lutein, lycopene, and astaxanthin, can absorb specific wavelengths of UV radiation. Bacterioruberin (BR) is a rare C_50_ carotenoid pigment belonging to the xanthophyll family, predominantly found in halophilic archaea—microorganisms that thrive in extremely salty environments. The additional carbon atoms and hydroxyl groups in bacterioruberin confer increased polarity, thereby enhancing its interaction with cell membranes and aqueous environments. Its rigid, elongated structure further contributes to its stronger anti-oxidant and photoprotective properties.

Carotenoids dissipate the absorbed energy as harmless heat, thereby reducing the available energy to initiate photo-induced molecular damage. Their chromophoric structure with extended conjugated double bonds enables them to function as biological filters, thereby reducing the penetration of high-energy photons into skin tissues. Carotenoids provide indirect photoprotection by neutralizing UV-induced reactive oxygen species (ROS), including singlet oxygen, superoxide anions, and hydrogen peroxide, all of which contribute to lipid peroxidation, DNA strand breaks, and matrix degradation. These ROS activate pro-aging signaling pathways, such as MAPK, NF-κB, and AP-1, leading to the upregulation of matrix metalloproteinases (e.g., MMP-1 and MMP-9) that degrade collagen and elastin. Carotenoids suppress these pathways, thereby preserving the dermal structural integrity [35,36,95].

Regular consumption of carotenoid-rich foods and oral supplementation have been shown to increase carotenoid concentrations in the skin, resulting in enhanced resistance to UVB-induced erythema, delayed onset of sunburn, and reduced markers of inflammation and DNA photodamage. Similarly, topical application of carotenoid-based formulations has demonstrated efficacy in ameliorating photodamage and improving skin tone and elasticity [37,96,97].

Carotenoids also attenuate UVA-induced pigmentation and oxidative stress, which are the major contributors to chronic photoaging and melanogenesis. Their accumulation in the epidermis and dermis forms a physiological anti-oxidant reservoir, reflecting dietary habits and the overall anti-oxidant status. Noninvasive techniques, such as resonance Raman spectroscopy, can quantify skin carotenoid levels and serve as biomarkers of skin health and dietary quality. In addition, epidemiological and interventional studies have suggested that individuals with elevated systemic levels of carotenoids exhibit fewer and shallower wrinkles, improved dermal hydration, and enhanced elasticity, underscoring the clinical relevance of carotenoids in anti-photoaging strategies [38,39,98,99].

The synergistic incorporation of carotenoids into sunscreens, serum, and oral nutraceuticals may offer a multifaceted defense against solar damage, particularly in populations at high risk of UV exposure. Their use signifies a paradigm shift from passive UV shielding to active dermal protection at both cellular and molecular levels.

#### 1.7.3. Anti-Inflammatory Properties of Carotenoids

Chronic low-grade inflammation, also known as “inflammaging,” is increasingly being recognized as a key contributor to cutaneous aging and the pathogenesis of numerous skin disorders, including atopic dermatitis, psoriasis, and photoaging-related degeneration. Carotenoids possess robust anti-inflammatory properties that enable them to modulate pathogenic mechanisms, thereby supporting dermal homeostasis and immune resilience. At the molecular level, carotenoids downregulate pro-inflammatory signaling pathways, most notably nuclear factor kappa B (NF-κB) and mitogen-activated protein kinases (MAPKs), both of which are critical mediators of cytokine transcription, leukocyte recruitment, and tissue injury. By inhibiting NF-κB activation, carotenoids suppress the expression of key inflammatory mediators, including tumor necrosis factor-alpha (TNF-α), interleukin-6 (IL-6), and interleukin-1 beta (IL-1β). Carotenoids inhibit the enzymatic activity of cyclooxygenase-2 (COX-2) and lipoxygenase (LOX), which are enzymes involved in the synthesis of pro-inflammatory eicosanoids such as prostaglandins and leukotrienes. This enzymatic suppression reduces vasodilation, erythema, edema, and leukocyte infiltration, all of which contribute to tissue damage and aging [100,101]. Another mechanism involves the modulation of macrophage polarization. Carotenoids facilitate the phenotypic switch from pro-inflammatory M1 macrophages to the anti-inflammatory M2 phenotype, which is associated with tissue repair, fibrosis regulation, and resolution of inflammation [102,103,104,105].

Astaxanthin, lutein, and beta-carotene have shown particular efficacy in reducing inflammatory markers in both systemic and cutaneous models and appear to influence cytokine production, as well as microRNA expression and epigenetic regulation of immune genes [106,107].

Additionally, carotenoids enhance skin immunity by modulating dendritic cell function and T cell activation, thereby contributing to a more balanced immune response in the skin microenvironment. This immunoregulatory function is critical for preventing chronic inflammatory damage and the associated dermal matrix degradation. The anti-inflammatory benefits of carotenoids are especially pronounced when delivered in synergistic combinations with other anti-oxidants (e.g., vitamins C and E and flavonoids), which collectively enhance cytokine suppression and oxidative defense [108,109,110].

Carotenoids exert multifactorial anti-inflammatory effects through inhibition of inflammatory enzymes, downregulation of cytokine expression, immune cell modulation, and anti-oxidant synergy. These mechanisms make carotenoids promising candidates for integrative approaches to treat inflammatory skin disorders and age-related cutaneous decline. In addition to neutralizing free radicals and anti-inflammatory effects, carotenoids also contribute to the skin structure by stimulating collagen production.

#### 1.7.4. Supporting Collagen Production

Collagen is the most abundant structural protein in the dermis, accounting for approximately 70–80% of the dry weight of the skin, and plays a central role in maintaining cutaneous firmness, elasticity, and tensile strength. Aging disrupts the homeostatic balance between collagen synthesis and degradation, resulting in reduced type I and III collagen levels, fiber fragmentation, and loss of dermal volume, which clinically manifests as wrinkles and sagging. Carotenoids, particularly β-carotene, lutein, astaxanthin, and lycopene, have been shown to enhance collagen synthesis and inhibit collagen breakdown, thereby preserving dermal architecture [111,112,113]. Their actions are mediated through multiple molecular mechanisms, including the following:

1. Upregulation of transforming growth factor-beta (TGF-β), a key growth factor that stimulates fibroblast proliferation and promotes expression of collagen genes.
2. Matrix metalloproteinases (MMPs), particularly MMP-1 (collagenase) and MMP-9 (gelatinase), are responsible for extracellular matrix (ECM) degradation during both intrinsic and extrinsic aging processes.
3. Stimulation of fibroblast activity, including increased migration, contractility, and secretion of collagen and elastin, enhances extracellular matrix remodeling and dermal regeneration.

Additionally, carotenoids improve fibroblast resistance to oxidative stress and inflammation, which otherwise impairs their collagen-synthesizing capabilities. For example, astaxanthin has demonstrated a significant ability to preserve fibroblast viability and boost type I collagen expression under ultraviolet (UV) exposure. Carotenoids may also affect the gene expression profiles associated with ECM turnover, including those involved in collagen biosynthesis, cross-linking enzymes such as lysyl oxidase, and structural glycoproteins such as fibronectin. Carotenoids have been found to inhibit the activity of inflammatory cytokines such as IL-1β and TNF-α, which are known to stimulate MMPs and accelerate collagen breakdown in aging or UV-damaged skin [53,54,114,115].

Carotenoids support collagen production by activating pro-fibrotic signaling pathways, preserving fibroblast function, reducing MMP-mediated degradation, and mitigating oxidative and inflammatory insults. These properties significantly contribute to their anti-wrinkle, firming, and dermo-reparative potential, underscoring their value in both nutritional and topical interventions for skin rejuvenation.

Carotenoids enhance skin hydration through two main mechanisms. First, they regulate aquaporins, which are proteins that control water movement within the skin. Second, they boost the production of hyaluronic acid, a molecule essential for retaining moisture.

#### 1.7.5. Synthesis of Hyaluronic Acid

Hyaluronic acid (HA) is a high-molecular-weight glycosaminoglycan abundantly present in the extracellular matrix (ECM) of the dermis, where it plays a critical role in water retention, tissue hydration, turgor, and mechanical resilience. The content and molecular integrity of HA decline progressively with age, contributing to dryness, reduced elasticity, and formation of fine lines and wrinkles [57,116,117].

Carotenoids have been shown to indirectly promote HA biosynthesis by modulating the inflammatory, oxidative, and growth factor signaling pathways that govern ECM metabolism. In particular, carotenoids, such as lutein, zeaxanthin, and beta-carotene, exert regulatory effects on the following:

− Cytokine expression, including the inhibition of pro-inflammatory mediators (e.g., IL-1β and TNF-α), is known to downregulate hyaluronan synthase (HAS) expression and accelerate HA degradation.
− The activation of growth factors, especially transforming growth factor-beta (TGF-β), a known stimulator of HAS-2 and HAS-3, is the key isoenzyme responsible for HA synthesis in dermal fibroblasts.
− Reduction in oxidative stress accelerates HA catabolism via ROS-mediated degradation and upregulation of hyaluronidases.

Although direct evidence for carotenoids stimulating HA synthesis remains limited, experimental models suggest that their anti-oxidative and anti-inflammatory properties create a biochemical environment conducive to HA production and stability. Carotenoids also appear to support fibroblast function by promoting the synthesis of HA, collagen, and elastin, thus contributing to global dermal ECM restoration. Carotenoids may inhibit matrix metalloproteinases, such as MMP-2 and MMP-3, as well as hyaluronidase enzymes, thereby preserving endogenous HA content and delaying its degradation [118,119,120].

In the context of cosmeceutical applications, combined delivery systems of HA and carotenoids, either topically or orally, may exert synergistic effects, improving skin hydration, elasticity, and barrier function. This synergy enhances moisturization and reinforces the cutaneous structure, all of which contribute to the youthful skin phenotype. While more research is required to define the precise molecular interplay between carotenoids and HA metabolism, current evidence strongly supports the inclusion of carotenoids in skin hydration strategies, particularly for anti-aging, barrier repair, and dermal regenerative purposes.

#### 1.7.6. Moisturizing the Skin

Optimal skin hydration is the cornerstone of cutaneous health and aesthetic appearance, and its decline with age contributes to xerosis, fine wrinkling, and compromised barrier function. With aging, the skin experiences a decline in its ability to retain water owing to decreased lipid synthesis, reduced natural moisturizing factors (NMFs), and impaired barrier integrity, making it more prone to xerosis, irritation, and mechanical fragility. Cutaneous hydration is essential to maintain epidermal elasticity, enzymatic activity, protection, and overall skin vitality [121,122].

Certain carotenoids (most notably, β-carotene, lutein, and astaxanthin) have been shown to enhance skin moisturization through several interrelated mechanisms, including barrier-enhancing, anti-inflammatory, and anti-oxidant mechanisms. By preserving intercellular lipid integrity, carotenoids help maintain the semi-permeability of the epidermis and prevent excessive water evaporation, which is a common feature of aged or damaged skin. In addition, carotenoids may stimulate fibroblasts to increase hyaluronic acid (HA) production, particularly under conditions of oxidative stress, by modulating cytokine profiles and enhancing the expression of HAS enzymes. This action indirectly promotes the water-binding capacity in the dermis, thereby supporting tissue turgor and pliability [123,124].

Carotenoids also influence the expression of aquaporins, particularly AQP3, which is a membrane water channel involved in epidermal water transport and glycerol permeability. Restoration of AQP3 function has been associated with improved skin hydration and elasticity, suggesting that carotenoids may facilitate water retention via modulation of membrane proteins [21,22,84,85]. Pro-inflammatory cytokines such as TNF-α and IL-1β can disrupt the function of fibroblasts and keratinocytes, impairing their ability to produce hyaluronic acid and epidermal lipids. By attenuating inflammatory signaling, carotenoids preserve the biosynthetic capacity of these cells and contribute to barrier repair and maintenance of hydration [125,126]. Carotenoids may enhance the activity of growth factors such as epidermal growth factor (EGF) and TGF-β, which regulate keratinocyte proliferation, matrix renewal, and dermal regeneration, which are all essential processes for maintaining hydrated and resilient skin surfaces [127,128,129,130].

Topical and dietary carotenoids have shown beneficial effects in increasing skin hydration, particularly when combined with essential fatty acids and polyphenols, which further strengthen the skin barrier and moisture-retention capacity. They have been associated with improved skin softness, elasticity, and surface smoothness, which are indirect hydration markers. Topically applied carotenoids act locally to support lipid organization, whereas dietary intake ensures systemic delivery to the dermal and epidermal layers. In addition to these bioactive strategies, adequate systemic hydration is also essential. Consumption of water-rich foods and anti-oxidant-rich beverages, such as green tea, enhances hydration and provides photoprotection and anti-inflammatory benefits. Well-hydrated skin exhibits greater elasticity, reduced wrinkle formation, and improved resilience to environmental stressors [131,132,133].

Carotenoids support skin moisturization by reinforcing the stratum corneum barrier, regulating water channels, preserving ECM integrity, and reducing pro-dehydration inflammatory signaling. The biochemical pathways activated by carotenoids ultimately lead to visible improvements in skin texture, reduced signs of aging, and enhanced skin resilience to environmental stressors [134,135].

To understand the systemic effects of carotenoids on skin health, it is essential to explore nutrient absorption, focusing on how these lipophilic molecules are integrated into the body through the gastrointestinal system.

### 1.8. Gut Health and Nutrient Absorption

Gut health plays a fundamental role in nutrient absorption, immune regulation, and systemic homeostasis, all of which directly influence skin physiology and anti-oxidant delivery efficacy. The intestinal microbiota, a dynamic and complex microbial ecosystem, modulates the digestion, transformation, and absorption of dietary compounds, including carotenoids [136,137,138]. Carotenoids are lipophilic molecules, the absorption of which depends on their incorporation into mixed micelles formed in the small intestine in the presence of bile salts and dietary lipids. These micelles facilitate passive diffusion of carotenoids across the intestinal epithelium, where they are subsequently packaged into chylomicrons for systemic transport. The bioavailability of carotenoids varies widely and is influenced by factors such as food matrix composition, fat co-ingestion, host genetics, and gut microbial composition [139,140,141]. Certain probiotic strains, such as Lactobacillus plantarum and Bifidobacterium breve, have been shown to enhance carotenoid bioconversion and intestinal uptake, likely through the production of bile salt hydrolases and the modulation of mucosal barrier integrity. Emerging studies have suggested that specific gut microbes facilitate the enzymatic cleavage of provitamin A carotenoids (e.g., β-carotene) into retinal and retinol, the bioactive forms of vitamin A that are critical for skin epithelial maintenance, immune function, and anti-oxidant defense [142,143,144].

Carotenoids may also influence gut microbial composition, exerting prebiotic-like effects that promote the growth of beneficial bacterial taxa (e.g., *Bifidobacterium and Faecalibacterium*) and suppress opportunistic pathogens. This bidirectional interaction between carotenoids and the microbiota contributes to intestinal barrier preservation and systemic immune modulation. Dysbiosis, characterized by disrupted microbial balance, has been associated with impaired carotenoid absorption, increased systemic inflammation, and a higher burden of oxidative stress, all of which can exacerbate premature skin aging. Thus, restoring microbial equilibrium through probiotic and prebiotic supplementation may enhance both nutrient bioefficacy and dermatological outcome. Additionally, the intestinal oxygen gradient, particularly physiological hypoxia in the lumen, plays a role in maintaining the barrier integrity and regulating nutrient transport. Recent findings indicate that hypoxia-responsive miRNAs, such as miRNA-320a, influence the expression of tight junction proteins and modulate epithelial permeability, thus affecting nutrient absorption and systemic exposure to bioactive compounds. Importantly, the efficiency of carotenoid assimilation is affected by the length and saturation of co-ingested fatty acids. Long-chain unsaturated fatty acids, such as oleic and linoleic acids, have been demonstrated to significantly improve micellarization efficiency, particularly for highly lipophilic carotenes such as lycopene and β-carotene [145,146,147,148].

The gut–skin axis represents a critical interface through which carotenoids influence dermatological health. Optimizing the gut microbiota composition, ensuring adequate fat intake, and correcting barrier dysfunction are essential strategies for enhancing carotenoid bioavailability and maximizing their protective effects on the skin.

#### 1.8.1. Unsaturated Fatty Acids and Carotenoid Bioavailability

The bioavailability of carotenoids, particularly their absorption in the small intestine, is highly dependent on their incorporation into mixed micelles, a process that is facilitated by the presence of dietary lipids and bile salts. Among these, unsaturated fatty acids (UFAs), especially monounsaturated fatty acids (MUFAs) and polyunsaturated fatty acids (PUFAs), play critical roles in enhancing carotenoid solubilization, transport, and intestinal uptake. Compared to saturated fatty acids (SFAs), UFAs form larger and more flexible micellar structures, which exhibit a superior capacity to incorporate lipophilic molecules such as β-carotene, lycopene, and lutein. This effect is particularly pronounced for carotenes, whose high hydrophobicity makes them more reliant on lipid-assisted solubilization [142,143].

Experimental studies have shown that oils high in unsaturated fatty acids, such as olive, sunflower, and soybean oils, can enhance carotenoid micellarization by two to three times compared to saturated fat sources, such as coconut or palm oil. This enhanced micellarization translates to higher postprandial plasma carotenoid levels and increased deposition in peripheral tissues, including the skin. The efficiency of micellar incorporation generally follows the order lutein/zeaxanthin > α-carotene/β-carotene > lycopene, reflecting differences in molecular polarity and structural rigidity. While the degree of unsaturation (mono- vs. polyunsaturated) does not appear to exert a consistently significant influence, the carbon chain length of the fatty acids is critical. Long-chain fatty acids (e.g., C18:1 oleic acid) promote the formation of larger, more stable micelles in contrast to medium-chain (C8) or short-chain (C4) fatty acids, which are less effective in facilitating carotenoid absorption. These mechanisms are particularly relevant for oral supplementation and dietary strategies for skin protection. Enhancing carotenoid bioaccessibility through co-ingestion with UFAs can significantly improve their systemic availability and dermal integration, thereby amplifying their anti-oxidant, photoprotective, and anti-inflammatory properties. Additionally, dietary emulsification techniques and lipid-based delivery systems (e.g., nanoemulsions, liposomes, and solid lipid nanoparticles) have been investigated to optimize carotenoid bioavailability. Such formulations often rely on unsaturated lipid matrices to improve gastrointestinal absorption and target tissue delivery [149,150,151,152].

The type, structure, and concentration of dietary fats play critical roles in modulating carotenoid bioavailability. Incorporating unsaturated fatty acids of appropriate chain length into diet or formulation significantly enhances intestinal absorption and skin-targeted anti-oxidant delivery, making them vital components in the nutritional modulation of skin aging.

#### 1.8.2. Oxidized Lipids and Anti-Oxidant Transport

Oxidized lipids are byproducts of lipid peroxidation, a process initiated by reactive oxygen species (ROS) that attack polyunsaturated fatty acids (PUFAs) within cell membranes. While excessive lipid peroxidation is typically viewed as detrimental to cell function and viability, recent studies have suggested that low to moderate levels of oxidized lipid species may modulate membrane dynamics in ways that influence anti-oxidant transport and signaling. During oxidative stress, lipid bilayers undergo structural remodeling, resulting in increased membrane fluidity and permeability. This biophysical alteration facilitates the transmembrane diffusion of lipophilic anti-oxidants, such as carotenoids, coenzyme Q10, and vitamin E (α-tocopherol), allowing them to more efficiently access intracellular compartments, particularly the mitochondria and endoplasmic reticulum, where ROS generation is highest [83,110].

A phenomenon known as the “whisker effect” has been described, in which oxidized lipid chains protrude into the aqueous phase, increasing the membrane flexibility and creating transient pores. These structural modifications might enhance vesicle fusion, endocytosis, and anti-oxidant penetration. While such adaptations may provide short-term compensatory benefits, chronic or excessive buildup of oxidized lipids, such as lipid hydroperoxides and malondialdehyde, leads to cytotoxicity, pro-inflammatory signaling, and ferroptotic cell death. The balance between oxidative damage and anti-oxidant defense is therefore critically regulated by detoxifying enzymes and lipid peroxidation suppressors [153,154].

The key players in this protective system include the following:

− Glutathione peroxidase 4 (GPX4): A selenoenzyme essential for reducing lipid hydroperoxides to non-toxic lipid alcohols, thereby protecting cellular membranes.
− Ferroptosis suppressor protein 1 (FSP1): Functions independently of GPX4 by recycling coenzyme Q10 into its reduced anti-oxidant form (ubiquinol), which prevents nonenzymatic lipid peroxidation in neutral lipid domains.
− Superoxide dismutase (SOD) and catalase (CAT): Reduce superoxide radicals and hydrogen peroxide, thereby mitigating the upstream drivers of lipid oxidation.

Additionally, vitamin E acts as a chain-breaking anti-oxidant that terminates lipid peroxidation cascades within the phospholipid bilayers. Coenzyme Q10, aside from its mitochondrial bioenergetic function, also protects against oxidative damage by preserving the membrane potential and supporting lipophilic anti-oxidant recycling. Oxidized lipids modulate anti-oxidant transport by altering membrane dynamics but also pose a significant risk for cellular dysfunction. Maintaining a balance between ROS production and detoxification mechanisms is essential for sustaining anti-oxidant efficacy and membrane integrity, particularly in tissues such as the skin, which are routinely exposed to oxidative challenges [155,156,157,158].

Although the benefits of carotenoids are well documented, the specific mechanisms through which they affect aging pathways in human skin are not yet fully understood. This review aimed to consolidate recent findings to shed light on these mechanisms.

## 2. Materials and Methods

### 2.1. Eligibility Criteria

#### 2.1.1. Inclusion Criteria

Studies were selected for inclusion if they met the following predefined criteria:

− The original research, clinical trials, and reviews have focused on the role of carotenoids in skin physiology and aging.
− Articles written in English and available in full-text format.
− Studies that provided mechanistic insights, clinical outcomes, or interventional results related to dietary or topical carotenoid application.

#### 2.1.2. Exclusion Criteria

Studies excluded if they met the following predefined criteria:

− Non-English articles;
− Studies unrelated to skin or focusing solely on non-carotenoid agents;
− Abstract-only publications or inaccessible sources.

### 2.2. Information Sources and Search Strategies

This narrative review was conducted according to the PRISMA 2020 guidelines. A comprehensive literature search was executed across six major electronic databases: PubMed, Scopus, Web of Science, Embase, Google Scholar, and the Cochrane Library. The search encompassed publications published from January 2000 to April 2025.

The following Medical Subject Headings (MeSH) terms and free-text keywords were used in various combinations: “carotenoids,” “skin aging,” “antioxidants,” “inflammation,” “photoprotection,” “collagen,” “hyaluronic acid,” “gut–skin axis,” “bioavailability,” “topical delivery,” and “lifestyle interventions.” Relevant articles were screened based on titles and abstracts, and the full texts were reviewed for inclusion. Manual cross-referencing of bibliographies of the included articles was also conducted to identify additional eligible sources.

### 2.3. Selection Process

Two independent researchers, C.S. and G.S., screened the study titles and abstracts, excluding duplicates and those deemed irrelevant. Full-text articles were obtained from the studies that successfully passed the initial screening. The same two authors subsequently conducted independent evaluations of these articles to ensure compliance with the eligibility criteria. Any discrepancies encountered during the screening process were addressed through discussions.

### 2.4. Data Collection Process and Data Items

All retrieved articles were independently screened by two reviewers, initially based on their titles and abstracts, followed by a comprehensive full-text evaluation to determine eligibility. Any discrepancies in the inclusion decisions were addressed through discussion and consensus. After applying these criteria, 176 articles were included in the final synthesis.

### 2.5. Study Risk of Bias Assessment

Quality assessment was conducted using the Risk of Bias 2 (RoB 2) tool as delineated in the Cochrane Handbook for Systematic Reviews of Interventions. Although the RoB 2 tool provides a comprehensive and structured framework for evaluating the risk of bias in randomized trials, its effective implementation requires meticulous planning and sufficient resources. This tool evaluates the risk of bias across five domains: (1) bias arising from the randomization process, (2) bias due to deviations from the intended interventions, (3) bias resulting from missing outcome data, (4) bias from the method of measuring the outcome, and (5) bias in the selection of the reported result. Two authors, C.S. and G.S., independently conducted the assessment, classifying each domain as “Low,” “Some Concerns,” or “High.” A study was considered to have an overall high risk of bias if one or more domains were rated as “High,” or if more than two domains were rated as having “Some Concerns.” Furthermore, studies with two domains rated as having “Some Concerns” were regarded as having some concerns for overall bias. Disagreements were resolved through discussion.

### 2.6. Effect Measures and Statistical Analysis

Key variables such as “carotenoids,” “skin aging,” “antioxidants,” “inflammation,” “photoprotection,” “collagen,” “hyaluronic acid,” “gut–skin axis,” “bioavailability,” “topical delivery,” and “lifestyle interventions” were extracted. Thematic synthesis was employed to organize the retrieved findings based on the mechanistic domain and protective role of carotenoids in skin aging. Particular emphasis was placed on studies reporting translational or clinical outcomes, including randomized controlled trials and interventional studies, where applicable. The narrative structure is designed to encompass a comprehensive range of topics, extending from a fundamental understanding of the skin structure and aging process to an in-depth examination of the mechanisms by which carotenoids exert their protective effects. These effects include anti-oxidative, anti-inflammatory, and photoprotective activities as well as their role in supporting collagen production and skin hydration. This review evaluates the clinical efficacy of both dietary and topical carotenoids in enhancing skin health and delineates future research directions in this domain. Although the benefits of carotenoids are well documented, the precise mechanisms by which they influence aging pathways in human skin remain incompletely understood. This review aims to synthesize recent research findings to advance the understanding of these mechanisms.

### 2.7. Study Selection

**Figure 4:**
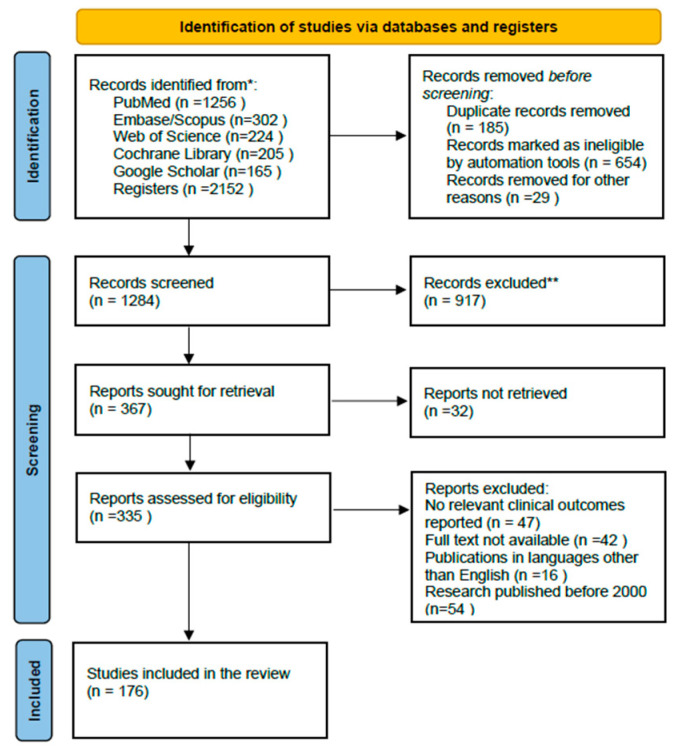
PRISMA flow diagram for identification of studies retrieved from databases.

The literature search identified 2152 articles across six major electronic databases: PubMed (n =1256), Embase/Scopus (n =302), Web of Science (n = 224), Cochrane Library (n =205), and Google Scholar (n = 165). Following the screening of titles and abstracts, 1802 articles were excluded, and the full texts of the remaining 335 articles were retrieved. Upon further analysis of adherence to the inclusion criteria, 176 studies were identified as eligible. The included studies were selected according to the process illustrated in the PRISMA flow diagram (Figure 4).

## 3. Results and Discussion

Carotenoids exhibit a broad spectrum of biological activities, and their contribution to human health extends beyond traditional anti-oxidant mechanisms. In dermatology and anti-aging medicine, carotenoids act as modulators of oxidative stress, inflammation, extracellular matrix remodeling, and immune response, thus influencing both the appearance and functional resilience of the skin. The ability of carotenoids to neutralize reactive oxygen species (ROS) is central to their anti-aging potential [159,160]. In addition to their anti-oxidant properties, these compounds exert regulatory effects on transcription factors (e.g., Nrf2 and NF-κB), enzyme systems (e.g., MMPs and COX-2), and cell signaling pathways (e.g., MAPK and IGF-1) that control cellular senescence and tissue regeneration. Notably, carotenoids such as lutein and zeaxanthin accumulate in specific tissues, such as the retina and skin, providing localized protection against photo-oxidative stress. In ophthalmology, their roles in preventing age-related macular degeneration (AMD) and cataracts have been well established. In dermatology, these compounds are increasingly studied for their roles in preserving collagen architecture, modulating pigmentation, and maintaining epidermal hydration [36,39,54,78]. Carotenoids can synergistically interact with other anti-oxidants to enhance defense mechanisms against oxidative stress, thereby promoting overall health and reducing the risk of chronic diseases [134,161].

Beyond the skin, carotenoids influence systemic health parameters:

1. Immune Function: Carotenoids enhance both innate and adaptive immunity by promoting antibody production, improving macrophage activity, and modulating cytokine secretion [100,101].
2. Respiratory Protection: Their anti-oxidant properties are beneficial for reducing inflammation in individuals exposed to air pollution or tobacco smoke, thereby supporting pulmonary function [33,34].
3. Cardiovascular Health: Lycopene and other carotenoids have been associated with improved endothelial function, reduced oxidative LDL, and a decreased risk of atherosclerosis [162].
4. Cancer Prevention: Some carotenoids exhibit pro-apoptotic and anti-proliferative effects in preclinical models of lung, prostate, and breast cancers, although clinical findings remain varied and dependent on dosage [147,163].
5. Neurocognitive Support: Some studies suggest a link between high dietary intake of carotenoids and cognitive benefits and reduced risk of neurodegenerative disorders, including Alzheimer’s and Parkinson’s disease, potentially through anti-inflammatory and neuroprotective mechanisms. It is important to note that research in this area is ongoing, and results have yet to conclusively establish causal relationships [60,61].
6. Metabolic Regulation: Carotenoids exert significant metabolic effects, including antilipidemic and antiglucemic properties. They enhance insulin sensitivity, reduce adiposity, and ameliorate markers of metabolic syndrome, thus indirectly benefiting skin health by improving systemic homeostasis [62].

In comparison to the more prevalent C_40_ carotenoids, such as β-carotene, lutein, lycopene, and astaxanthin, bacterioruberin demonstrates superior biological activities in several areas, including anti-oxidant, photoprotective, immunomodulatory, antitumoral, antilipidic, and membrane stability functions.

Depression is a major cause of disability worldwide and is characterized by persistent sadness, hopelessness, and other symptoms that can affect various aspects of life, including physical health. This can affect skin health and aging through a lack of interest in physical appearance and reduced self-esteem [164,165]. Physical appearance significantly influences the self-esteem of older adults, extending beyond skin health to affect various dimensions of psychological well-being and overall quality of life [166,167,168]. Individuals with chronic dermatological lesions are at elevated risks of depression and anxiety. Depression can intensify stress, leading to increased levels of cortisol and other stress-related hormones that have been correlated with chronic inflammation. Individuals with depression frequently demonstrate diminished leukocyte activity and modified cytokine profiles, which can impair immune function, leading to a slower and less efficient healing process [169,170].

The relationship between carotenoids and mood regulation is an emerging area of interest. Epidemiological data indicate a connection between higher carotenoid intake and lower incidence of depressive symptoms, potentially mediated by reduced systemic inflammation, oxidative stress, and enhanced neurotransmitter function [59,63]. This is especially relevant to aging populations, in which psychodermatological disorders may influence self-care, appearance-related self-esteem, and skin aging trajectories. Sleep disturbances, which impair circadian rhythm and skin barrier function, are exacerbated by aging [171]. Carotenoids may indirectly support circadian entrainment through their effects on pineal melatonin synthesis, especially when co-supplemented with nutrients that influence sleep quality [27,28].

Additionally, in individuals with diabetes, elevated levels of oxidative stress and reduced anti-oxidant enzyme activity compromise skin integrity, accelerate aging, and impair wound healing. Advanced glycation end products (AGEs) accumulate in the dermis, altering collagen cross-linking, and elastin structure. Carotenoids may mitigate these effects through their anti-glycation, free radical scavenging, and anti-inflammatory actions [17,18].

Chronic exposure to environmental stressors such as UV radiation, airborne pollutants, and toxins activates multiple pro-aging signaling cascades, including the p53, AhR, and MAPK pathways, which lead to DNA damage, cellular apoptosis, and immunosuppression. Carotenoids help maintain the stability of genetic material by blocking certain damaging pathways both directly and indirectly [100,101].

Carotenoids operate as multifunctional bioactive compounds with dermatological, systemic, and neuroendocrine effects. Their integration into preventive strategies, dietary regimens, and topical formulations represents a promising and evolving frontier in the field of healthy aging. For example, although studies have demonstrated that both oral and topical application of carotenoids can effectively protect the skin, the combined method seems to offer the strongest anti-oxidant protection [83].

Lifestyle modifications are essential for maintaining skin health and can significantly enhance the effectiveness of various therapeutic strategies. These modifications interact with diverse treatment methodologies in multiple ways, offering a comprehensive approach to skincare and the management of dermatological conditions. Research indicates that lifestyle factors, including nutrition, sleep, physical activity, and social interactions, contribute to the enhancement of skin health and overall well-being with age. These factors can influence the skin microbiome, which plays a crucial role in maintaining the epithelial barrier function, safeguarding against pathogens, and interacting with the immune system. The composition of a healthy skin microbiome is determined by both genetic and environmental factors, including lifestyle [137,172].

Integrating lifestyle changes with traditional medical treatments can improve the outcomes of various dermatological conditions, thereby promoting skin health. Some lifestyle modifications that can enhance the benefits of carotenoids for skin health are as follows:

1. Dietary Changes

Increased intake of carotenoid-rich foods: Consuming a diet rich in fruits and vegetables high in carotenoids can enhance skin protection against environmental stressors and enhance well-being. Foods such as tomatoes (rich in lycopene), carrots, sweet potatoes, and leafy greens (rich in beta-carotene, lutein, and zeaxanthin) should be included in the diet. In postmenopausal women, increased carotenoid intake is associated with decreased levels of depression [62,134,173].

Balanced nutrition: A diet that supplies a diverse array of carotenoids can maximize their synergistic effects in skin protection. Carotenoids from various sources may function together to provide extensive anti-oxidant benefits [26,82].

2. Combination of Oral and Topical Carotenoids

Carotenoids, both orally and topically, enhanced the protective effects on the skin. Oral intake boosts systemic anti-oxidant activity, whereas topical application directly improves skin resilience to UV exposure [37,39,41,42].

3. Avoidance of tobacco and reduced exposure to UV radiation

Smoking decreases skin carotenoid levels owing to increased oxidative stress. Avoiding smoking and reducing UV exposure through protective clothing and sunscreens can prevent the depletion of carotenoids, allowing them to function more effectively as anti-oxidants [32,34].

4. Optimal Carotenoid Levels

Maintaining optimal carotenoid levels is important because excessive intake can lead to pro-oxidative effects rather than anti-oxidant benefits [44,119].

5. Routine Monitoring and Supplementation

Regular monitoring of skin carotenoid levels can help in tailoring dietary interventions. Supplements might be considered if dietary intake is insufficient, although current recommendations suggest obtaining carotenoids primarily through whole foods where possible [46,145].

By making these lifestyle adjustments, individuals can enhance the effectiveness of carotenoids in protecting the skin from harmful external factors and maintaining skin health.

Carotenoids enhance skin health by quenching singlet oxygen and neutralizing free radicals, which can otherwise cause oxidative damage. These protective effects are attributed to their ability to inhibit processes, such as lipid peroxidation in human cells, thereby offering defense against UV-induced oxidative damage. Moreover, lycopene has shown promise as an oral sun protectant, protecting the skin against UV-induced damage and contributing to the maintenance of overall skin health. Studies indicate that carotenoids possess photoprotective effects not only by directly absorbing light but also by scavenging reactive oxygen species (ROS) and modulating UV-induced gene expression and stress-related signaling mechanisms. They have been shown to improve skin parameters such as elasticity, hydration, texture, and appearance of wrinkles and age spots through dietary intake, particularly in interventions that include carotenoid-rich foods [85,87,89,124].

### 3.1. Controversies Surrounding Carotenoids in Skin Aging

Despite these benefits, there are ongoing debates regarding the ideal dosage and delivery method for carotenoids in skincare. Under specific conditions, particularly at elevated concentrations, carotenoids may exhibit pro-oxidative effects rather than their typical protective properties, potentially resulting in detrimental effects on skin. This dual functionality underscores the necessity of optimizing the balance between the anti-oxidant and pro-oxidant activities of carotenoids for dermatological applications. The adverse effects of excessive carotenoid accumulation are equivalent to the negative consequences of dietary elimination [174,175].

Ongoing research is essential to clarify dose–response relationships for various carotenoids in both systemic and topical applications and to determine optimal delivery formats, especially in multi-compound formulations.

Carotenoids are vital anti-oxidants that bolster the skin’s defense against environmental damage, mitigate oxidative stress, and support healthy skin aging and management of chronic diseases. However, their role in preventing skin photocarcinogenesis has not yet been established. Recent research indicates that carotenoids may hold promise as therapeutic agents against non-melanoma skin cancers. However, further empirical evidence is necessary to validate their efficacy in preventing photocarcinogenesis [88,96,147,176].

Continued research is crucial for understanding the interactions between carotenoids, microbiota, and host signaling pathways in the context of skin aging and disease modulation.

High doses of β-carotene have been associated with an elevated risk of cancer in individuals who smoke or have been exposed to asbestos, prompting concerns regarding indiscriminate supplementation [96,109,147]. Excessive consumption may result in carotenodermia, a benign yet cosmetically undesirable discoloration of the skin. Prolonged use may also induce pigmentation disorders or allergic reactions, although these effects are infrequent. There is no consensus on the optimal dosage, formulation, or combination of carotenoids for promoting skin health. While carotenoids hold promise in the fight against skin aging, their application is not without controversy. Future research should aim to clarify dosage thresholds, mechanisms of action, and long-term safety, especially in vulnerable populations. A balanced perspective is essential to guide both clinical practice and consumer use.

### 3.2. Strengths and Limitations of the Study

This review provides a comprehensive synthesis of current knowledge regarding the role of carotenoids in skin aging, integrating both mechanistic and clinical perspectives. A notable strength lies in its coverage of multiple carotenoid compounds—including β-carotene, lycopene, lutein, astaxanthin, and emerging molecules such as bacterioruberin—highlighting their diverse anti-oxidant and anti-inflammatory properties. The discussion of molecular pathways, particularly those involving oxidative stress and cellular signaling (e.g., MAPK, Nrf2, NF-κB), offers valuable mechanistic insights that support the therapeutic potential of carotenoids in dermatology. Furthermore, the dual focus on topical and oral applications enhances the clinical relevance of the findings.

However, several limitations must be acknowledged. The review is based on secondary data and does not present original experimental results, which may limit the depth of interpretation. In addition, variability among the cited studies—such as differences in dosage, duration, and population characteristics—poses challenges for drawing consistent conclusions. The long-term safety profile of carotenoid supplementation, including potential adverse effects like carotenodermia, their role in preventing skin photocarcinogenesis, or pro-oxidant activity at high concentrations, remains insufficiently explored. Moreover, issues related to bioavailability and tissue distribution are only briefly addressed, despite their importance in clinical efficacy. Finally, the absence of standardized guidelines for carotenoid intake specific to skin health underscores the need for further research and regulatory clarity.

## 4. Conclusions

Skin aging is driven by a complex interplay between intrinsic genetic and hormonal factors and extrinsic environmental influences, resulting in oxidative stress, chronic inflammation, mitochondrial dysfunction, and dermal matrix degradation. Carotenoids, with their diverse biological properties, present promising opportunities to mitigate these processes at both the molecular and tissue levels.

This review highlights that carotenoids act through a synergistic array of mechanisms, including anti-oxidant activity, photoprotection, inhibition of pro-inflammatory mediators, stimulation of collagen and hyaluronic acid synthesis, and support of barrier integrity and hydration. Their protective roles extend beyond the skin and influence systemic immune function, metabolic health, neurocognitive stability, and mood regulation, all of which indirectly contribute to dermatological homeostasis. Cardiovascular diseases can manifest in the skin through mechanisms such as impaired circulation, chronic inflammation, or cholesterol accumulation. Diabetes mellitus impacts the skin via chronic hyperglycemia, microvascular damage, and neuropathy, leading to conditions such as diabetic dermopathy. While depression does not directly cause skin lesions, it can affect the skin through psychosomatic and behavioral pathways, such as psychogenic pruritus and neglect of personal care. In Parkinson’s disease, autonomic nervous system dysfunction results in oily skin, with either excessive or reduced sweating. In Alzheimer’s disease, the skin becomes fragile and susceptible to injuries due to immobility and insufficient care. The skin serves as a “barometer” of internal health, with changes potentially indicating early signs or complications of systemic diseases. Therefore, vigilant monitoring and collaboration between dermatologists and other specialists, including cardiologists, endocrinologists, neurologists, and psychiatrists, are crucial.

Combining carotenoid-rich nutrition with evidence-based cosmeceutical applications and lifestyle interventions such as regular physical activity, stress reduction, adequate sleep, and microbiome modulation has emerged as a holistic strategy for promoting healthy skin aging. This integrated approach enhances therapeutic outcomes, minimizes the risk of adverse effects, and supports long-term skin resilience. The incorporation of unsaturated dietary fats, probiotics, and advanced delivery systems can significantly improve carotenoid bioavailability and dermal targeting, especially in populations with an increased oxidative burden or impaired absorption, such as the elderly or individuals with metabolic disorders.

Carotenoids are a scientifically validated, safe, and versatile class of compounds that can be strategically leveraged to prevent premature skin aging, enhance repair processes, and maintain cutaneous health. Their integration into personalized skincare and nutritional protocols supports the development of precise anti-aging strategies based on molecular and clinical evidence.

Despite their protective roles, controversies exist concerning the optimal dosage and delivery method for carotenoids in skincare. Additionally, although carotenoids provide notable UV protection, their effectiveness as photoprotectors and their role in reducing skin cancer risk remain uncertain. Current research shows that carotenoids are promising agents against non-melanoma skin cancer; however, their ability to prevent photocarcinogenesis has not been conclusively proven.

In conclusion, although certain studies support the protective role of carotenoids in mitigating skin aging, the debate persists regarding their exact mechanisms, optimal delivery methods, dosages, potential pro-oxidative effects, and roles in chemoprevention. Further research is essential to clarify these areas and establish standardized guidelines for clinical application.

## Data Availability

Not applicable.

## References

[1] Stamatas G.N., Roux P.F., Boireau-Adamezyk E., Lboukili I., Oddos T. (2023). Skin maturation from birth to 10 years of age: Structure, function, composition, and microbiome. Exp. Dermatol..

[2] Choi E.H. (2025). Skin Barrier Function in Neonates and Infants. Allergy Asthma Immunol. Res..

[3] de Szalay S., Wertz P.W. (2023). Protective Barriers Provided by the Epidermis. Int. J. Mol. Sci..

[4] Dingwall H.L., Tomizawa R.R., Aharoni A., Hu P., Qiu Q., Kokalari B., Martinez S.M., Donahue J.C., Aldea D., Mendoza M. (2024). Sweat gland development requires an eccrine dermal niche and couples two epidermal programs. Dev. Cell.

[5] Stevenson S., Thornton J. (2007). Effect of estrogens on skin aging and the potential role of SERMs. Clin. Interv. Aging.

[6] Bravo B., Penedo L., Carvalho R., Dal Vesco C., Calomeni M., Gapanowicz D., Kemen E., Paes R., Renke G. (2024). Dermatological Changes during Menopause and HRT: What to Expect?. Cosmetics.

[7] Nedelec B., Forget N.J., Hurtubise T., Cimino S., de Muszka F., Legault A., Liu W.L., de Oliveira A.C.M.T.G., Calva V., Correa J.A.A. (2016). Skin characteristics: Normative data for elasticity, erythema, melanin, and thickness at 16 different anatomical locations. Ski. Res. Technol..

[8] Ganceviciene R., Liakou A.I., Theodoridis A., Makrantonaki E., Zouboulis C.C. (2012). Skin anti-aging strategies. Dermatoendocrinol..

[9] Lee H., Hong Y., Kim M. (2021). Structural and Functional Changes and Possible Molecular Mechanisms in Aged Skin. Int. J. Mol. Sci..

[10] Papaccio F., D’Arino A., Caputo S., Bellei B. (2022). Focus on the Contribution of Oxidative Stress in Skin Aging. Antioxidants.

[11] Rinnerthaler M., Bischof J., Streubel M.K., Trost A., Richter K. (2015). Oxidative Stress in Aging Human Skin. Biomolecules.

[12] Tu Y., Quan T. (2016). Oxidative Stress and Human Skin Connective Tissue Aging. Cosmetics.

[13] Hajam Y.A., Rani R., Ganie S.Y., Sheikh T.A., Javaid D., Qadri S.S., Pramodh S., Alsulimani A., Alkhanani M.F., Harakeh S. (2022). Oxidative Stress in Human Pathology and Aging: Molecular Mechanisms and Perspectives. Cells.

[14] Wedel S., Martic I., Navarro L.G., Ploner C., Pierer G., Jansen-Dürr P., Cavinato M. (2023). Depletion of growth differentiation factor 15 (GDF15) leads to mitochondrial dysfunction and premature senescence in human dermal fibroblasts. Aging Cell.

[15] Li P., Lv H., Zhang B., Duan R., Zhang X., Lin P., Song C., Liu Y. (2022). Growth Differentiation Factor 15 Protects SH-SY5Y Cells from Rotenone-Induced Toxicity by Suppressing Mitochondrial Apoptosis. Front. Aging Neurosci..

[16] Gkogkolou P., Böhm M. (2012). Advanced glycation end products: Key players in skin aging?. Dermato-Endocrinology.

[17] Zhang Y., Zhang Z., Tu C., Chen X., He R. (2025). Advanced Glycation End Products in Disease Development and Potential Interventions. Antioxidants.

[18] Fournet M., Bonté F., Desmoulière A. (2018). Glycation Damage: A Possible Hub for Major Pathophysiological Disorders and Aging. Aging Dis..

[19] Chen C.-Y., Zhang J.-Q., Li L., Guo M.-M., He Y.-F., Dong Y.-M., Meng H., Yi F. (2022). Advanced Glycation End Products in the Skin: Molecular Mechanisms, Methods of Measurement, and Inhibitory Pathways. Front. Med..

[20] Karimi N., Ahmadi V. (2024). Aquaporin Channels in Skin Physiology and Aging Pathophysiology: Investigating Their Role in Skin Function and the Hallmarks of Aging. Biology.

[21] Hara M., Ma T., Verkman A.S. (2002). Selectively Reduced Glycerol in Skin of Aquaporin-3-deficient Mice May Account for Impaired Skin Hydration, Elasticity, and Barrier Recovery. J. Biol. Chem..

[22] Tricarico P.M., Mentino D., De Marco A., Del Vecchio C., Garra S., Cazzato G., Foti C., Crovella S., Calamita G. (2022). Aquaporins Are One of the Critical Factors in the Disruption of the Skin Barrier in Inflammatory Skin Diseases. Int. J. Mol. Sci..

[23] Vrettou C.S., Issaris V., Kokkoris S., Poupouzas G., Keskinidou C., Lotsios N.S., Kotanidou A., Orfanos S.E., Dimopoulou I., Vassiliou A.G. (2024). Exploring Aquaporins in Human Studies: Mechanisms and Therapeutic Potential in Critical Illness. Life.

[24] Meli R., Pirozzi C., Pelagalli A. (2018). New Perspectives on the Potential Role of Aquaporins (AQPs) in the Physiology of Inflammation. Front. Physiol..

[25] Juncan A.M., Moisă D.G., Santini A., Morgovan C., Rus L.-L., Vonica-Țincu A.L., Loghin F. (2021). Advantages of Hyaluronic Acid and Its Combination with Other Bioactive Ingredients in Cosmeceuticals. Molecules.

[26] Siquier-Dameto G., Boadas-Vaello P., Verdú E. (2024). Intradermal Treatment with a Hyaluronic Acid Complex Supplemented with Amino Acids and Antioxidant Vitamins Improves Cutaneous Hydration and Viscoelasticity in Healthy Subjects. Antioxidants.

[27] Slominski A.T., Hardeland R., Zmijewski M.A., Slominski R.M., Reiter R.J., Paus R. (2018). Melatonin: A Cutaneous Perspective on its Production, Metabolism, and Functions. J. Investig. Dermatol..

[28] Kleszczynski K., Fischer T.W. (2012). Melatonin and human skin aging. Dermato-Endocrinology.

[29] Bocheva G., Slominski R.M., Janjetovic Z., Kim T.-K., Böhm M., Steinbrink K., Reiter R.J., Kleszczyński K., Slominski A.T. (2022). Protective Role of Melatonin and Its Metabolites in Skin Aging. Int. J. Mol. Sci..

[30] Rusanova I., Martínez-Ruiz L., Florido J., Rodríguez-Santana C., Guerra-Librero A., Acuña-Castroviejo D., Escames G. (2019). Protective Effects of Melatonin on the Skin: Future Perspectives. Int. J. Mol. Sci..

[31] Farris P.K., Valacchi G. (2022). Ultraviolet Light Protection: Is It Really Enough?. Antioxidants.

[32] Parrado C., Mercado-Saenz S., Perez-Davó A., Gilaberte Y., Gonzalez S., Juarranz A. (2019). Environmental Stressors on Skin Aging. Mechanistic Insights. Front. Pharmacol..

[33] Bocheva G., Slominski R.M., Slominski A.T. (2023). Environmental Air Pollutants Affecting Skin Functions with Systemic Implications. Int. J. Mol. Sci..

[34] Drakaki E., Dessinioti C., Antoniou C.V. (2014). Air pollution and the skin. Front. Environ. Sci..

[35] Zhang H., Dong Y., Xiao X., Cui X., Gu X. (2025). Omics-Based Interaction Analysis Reveals Interplay of Chemical Pollutant (Ozone) and Photoradiation (UVSSR) Stressors in Skin Damage. Biology.

[36] Ma Y., Li C., Su W., Sun Z., Gao S., Xie W., Zhang B., Sui L. (2025). Carotenoids in Skin Photoaging: Unveiling Protective Effects, Molecular Insights, and Safety and Bioavailability Frontiers. Antioxidants.

[37] Parrado C., Philips N., Gilaberte Y., Juarranz A., González S. (2018). Oral Photoprotection: Effective Agents and Potential Candidates. Front. Med..

[38] Flieger J., Raszewska-Famielec M., Radzikowska-Büchner E., Flieger W. (2024). Skin Protection by Carotenoid Pigments. Int. J. Mol. Sci..

[39] Sullivan M., Gonzalez Obezo C., Lipsky Z., Panchal A., Jensen J. (2025). Frontiers in Topical Photoprotection. Cosmetics.

[40] Jomova K., Raptova R., Alomar S.Y., Alwasel S.H., Nepovimova E., Kuca K., Valko M. (2023). Reactive oxygen species, toxicity, oxidative stress, and antioxidants: Chronic diseases and aging. Arch. Toxicol..

[41] Miazek K., Beton K., Śliwińska A., Brożek-Płuska B. (2022). The Effect of β-Carotene, Tocopherols and Ascorbic Acid as Anti-Oxidant Molecules on Human and Animal In Vitro/In Vivo Studies: A Review of Research Design and Analytical Techniques Used. Biomolecules.

[42] Rao M.J., Duan M., Zhou C., Jiao J., Cheng P., Yang L., Wei W., Shen Q., Ji P., Yang Y. (2025). Antioxidant Defense System in Plants: Reactive Oxygen Species Production, Signaling, and Scavenging During Abiotic Stress-Induced Oxidative Damage. Horticulturae.

[43] Zandi P., Schnug E. (2022). Reactive Oxygen Species, Antioxidant Responses and Implications from a Microbial Modulation Perspective. Biology.

[44] Stromsnes K., Correas A.G., Lehmann J., Gambini J., Olaso-Gonzalez G. (2021). Anti-Inflammatory Properties of Diet: Role in Healthy Aging. Biomedicines.

[45] Majdan M., Bobrowska-Korczak B. (2022). Active Compounds in Fruits and Inflammation in the Body. Nutrients.

[46] Darvin M.E., Sterry W., Lademann J., Vergou T. (2011). The Role of Carotenoids in Human Skin. Molecules.

[47] Cao C., Xiao Z., Wu Y., Ge C. (2020). Diet and Skin Aging—From the Perspective of Food Nutrition. Nutrients.

[48] Collins A.R. (2001). Carotenoids and genomic stability. Mutat. Res..

[49] Kaźmierczak-Barańska J., Boguszewska K., Karwowski B.T. (2020). Nutrition Can Help DNA Repair in the Case of Aging. Nutrients.

[50] Brand R.M., Wipf P., Durham A., Epperly M.W., Greenberger J.S., Falo L.D. (2018). Targeting Mitochondrial Oxidative Stress to Mitigate UV-Induced Skin Damage. Front. Pharmacol..

[51] Sharma P., Sampath H. (2019). Mitochondrial DNA Integrity: Role in Health and Disease. Cells.

[52] Qin Z., Fisher G.J., Voorhees J.J., Quan T. (2018). Actin cytoskeleton assembly regulates collagen production via TGF-β type II receptor in human skin fibroblasts. J. Cell. Mol. Med..

[53] Tracy L.E., Minasian R.A., Caterson E.J. (2016). Extracellular Matrix and Dermal Fibroblast Function in the Healing Wound. Adv. Wound Care.

[54] Shin J.-W., Kwon S.-H., Choi J.-Y., Na J.-I., Huh C.-H., Choi H.-R., Park K.-C. (2019). Molecular Mechanisms of Dermal Aging and Antiaging Approaches. Int. J. Mol. Sci..

[55] Boo Y.C. (2022). Ascorbic Acid (Vitamin C) as a Cosmeceutical to Increase Dermal Collagen for Skin Antiaging Purposes: Emerging Combination Therapies. Antioxidants.

[56] Wang K., Wen D., Xu X., Zhao R., Jiang F., Yuan S., Zhang Y., Gao Y., Li Q. (2023). Extracellular matrix stiffness-The central cue for skin fibrosis. Front. Mol. Biosci..

[57] Yue Z., Shao K. (2025). Visualization of the Relationship Between Hyaluronic Acid and Wound Healing: A Bibliometric Analysis. Ski. Res. Technol..

[58] Chylińska N., Maciejczyk M. (2025). Hyaluronic Acid and Skin: Its Role in Aging and Wound-Healing Processes. Gels.

[59] Rasmus P., Kozłowska E. (2023). Antioxidant and Anti-Inflammatory Effects of Carotenoids in Mood Disorders: An Overview. Antioxidants.

[60] Valotto Neto L.J., Reverete de Araujo M., Moretti Junior R.C., Mendes Machado N., Joshi R.K., dos Santos Buglio D., Barbalho Lamas C., Direito R., Fornari Laurindo L., Tanaka M. (2024). Investigating the Neuroprotective and Cognitive-Enhancing Effects of *Bacopa monnieri*: A Systematic Review Focused on Inflammation, Oxidative Stress, Mitochondrial Dysfunction, and Apoptosis. Antioxidants.

[61] Flieger J., Forma A., Flieger W., Flieger M., Gawlik P.J., Dzierżyński E., Maciejewski R., Teresiński G., Baj J. (2024). Carotenoid Supplementation for Alleviating the Symptoms of Alzheimer’s Disease. Int. J. Mol. Sci..

[62] Kalogerakou T., Antoniadou M. (2024). The Role of Dietary Antioxidants, Food Supplements and Functional Foods for Energy Enhancement in Healthcare Professionals. Antioxidants.

[63] Liu F., Bai Q., Tang W., Zhang S., Guo Y., Pan S., Ma X., Yang Y., Fan H. (2024). Antioxidants in neuropsychiatric disorder prevention: Neuroprotection, synaptic regulation, microglia modulation, and neurotrophic effects. Front. Neurosci..

[64] Tanprasertsuk J., Scott T.M., Barbey A.K., Barger K., Wang X.-D., Johnson M.A., Poon L.W., Vishwanathan R., Matthan N.R., Lichtenstein A.H. (2021). Carotenoid-Rich Brain Nutrient Pattern Is Positively Correlated with Higher Cognition and Lower Depression in the Oldest Old with No Dementia. Front. Nutr..

[65] Kunst C., Schmid S., Michalski M., Tümen D., Buttenschön J., Müller M., Gülow K. (2023). The Influence of Gut Microbiota on Oxidative Stress and the Immune System. Biomedicines.

[66] Heidari M., Maleki Vareki S., Yaghobi R., Karimi M.H. (2024). Microbiota activation and regulation of adaptive immunity. Front. Immunol..

[67] Amimo J.O., Michael H., Chepngeno J., Raev S.A., Saif L.J., Vlasova A.N. (2022). Immune Impairment Associated with Vitamin A Deficiency: Insights from Clinical Studies and Animal Model Research. Nutrients.

[68] Bhutta N.K., Xu X., Jian C., Wang Y., Liu Y., Sun J., Han B., Wu S., Javeed A. (2024). Gut microbiota mediated T cells regulation and autoimmune diseases. Front. Microbiol..

[69] Shanaida M., Mykhailenko O., Lysiuk R., Hudz N., Balwierz R., Shulhai A., Shapovalova N., Shanaida V., Bjørklund G. (2025). Carotenoids for Antiaging: Nutraceutical, Pharmaceutical, and Cosmeceutical Applications. Pharmaceuticals.

[70] Molteni C., La Motta C., Valoppi F. (2022). Improving the Bioaccessibility and Bioavailability of Carotenoids by Means of Nanostructured Delivery Systems: A Comprehensive Review. Antioxidants.

[71] Saini R.K., Prasad P., Lokesh V., Shang X., Shin J., Keum Y.-S., Lee J.-H. (2022). Carotenoids: Dietary Sources, Extraction, Encapsulation, Bioavailability, and Health Benefits—A Review of Recent Advancements. Antioxidants.

[72] Pereira A.G., Otero P., Echave J., Carreira-Casais A., Chamorro F., Collazo N., Jaboui A., Lourenço-Lopes C., Simal-Gandara J., Prieto M.A. (2021). Xanthophylls from the Sea: Algae as Source of Bioactive Carotenoids. Mar. Drugs.

[73] Andrés C.M.C., Pérez de la Lastra J.M., Juan C.A., Plou F.J., Pérez-Lebeña E. (2024). Antioxidant Metabolism Pathways in Vitamins, Polyphenols, and Selenium: Parallels and Divergences. Int. J. Mol. Sci..

[74] Pradel P., Calisto N., Navarro L., Barriga A., Vera N., Aranda C., Simpfendorfer R., Valdés N., Corsini G., Tello M. (2021). Carotenoid Cocktail Produced by An Antarctic Soil *Flavobacterium* with Biotechnological Potential. Microorganisms.

[75] Conboy Stephenson R., Ross R.P., Stanton C. (2021). Carotenoids in Milk and the Potential for Dairy Based Functional Foods. Foods.

[76] Iddir M., Porras Yaruro J.F., Cocco E., Hardy E.M., Appenzeller B.M.R., Guignard C., Larondelle Y., Bohn T. (2021). Impact of Protein-Enriched Plant Food Items on the Bioaccessibility and Cellular Uptake of Carotenoids. Antioxidants.

[77] Etcheverry P., Grusak M.A., Fleige L.E. (2012). Application of in vitro bioaccessibility and bioavailability methods for calcium, carotenoids, folate, iron, magnesium, polyphenols, zinc, and vitamins B_6_, B_12_, D, and E. Front. Physiol..

[78] Casagrande S., Dell’omo G., Costantini D., Tagliavini J., Groothuis T. (2011). Variation of a carotenoid-based trait in relation to oxidative stress and endocrine status during the breeding season in the Eurasian kestrel: A multi-factorial study. Comp. Biochem. Physiol. Part A Mol. Integr. Physiol..

[79] Ferrando B.O., Baenas N., Periago M.J. (2024). Changes in Carotenoids and Quality Parameters of Sweet Paprika (*Capsicum annuum*) After an Accelerated Heat Treatment. Antioxidants.

[80] Liu H., Cao X., Azam M., Wang C., Liu C., Qiao Y., Zhang B. (2022). Metabolism of Carotenoids and β-Ionone Are Mediated by Carotenogenic Genes and *PpCCD4* Under Ultraviolet B Irradiation and During Fruit Ripening. Front. Plant Sci..

[81] Yao R., Fu W., Du M., Chen Z.-X., Lei A.-P., Wang J.-X. (2022). Carotenoids Biosynthesis, Accumulation, and Applications of a Model Microalga *Euglenagracilis*. Mar. Drugs.

[82] Adamantidi T., Lafara M.-P., Venetikidou M., Likartsi E., Toganidou I., Tsoupras A. (2025). Utilization and Bio-Efficacy of Carotenoids, Vitamin A and Its Vitaminoids in Nutricosmetics, Cosmeceuticals, and Cosmetics’ Applications with Skin-Health Promoting Properties. Appl. Sci..

[83] de Souza Guedes L., Martinez R.M., Bou-Chacra N.A., Velasco M.V.R., Rosado C., Baby A.R. (2021). An Overview on Topical Administration of Carotenoids and Coenzyme Q10 Loaded in Lipid Nanoparticles. Antioxidants.

[84] Pincemail J., Meziane S. (2022). On the Potential Role of the Antioxidant Couple Vitamin E/Selenium Taken by the Oral Route in Skin and Hair Health. Antioxidants.

[85] Fiedor J., Burda K. (2014). Potential Role of Carotenoids as Antioxidants in Human Health and Disease. Nutrients.

[86] Milani A., Basirnejad M., Shahbazi S., Bolhassani A. (2017). Carotenoids: Biochemistry, pharmacology and treatment. Br. J. Pharmacol..

[87] Cantrell A., McGarvey D.J., Truscott T.G., Rancan F., Böhm F. (2003). Singlet oxygen quenching by dietary carotenoids in a model membrane environment. Arch. Biochem. Biophys..

[88] Black H.S., Boehm F., Edge R., Truscott T.G. (2020). The Benefits and Risks of Certain Dietary Carotenoids that Exhibit both Anti- and Pro-Oxidative Mechanisms—A Comprehensive Review. Antioxidants.

[89] Boehm F., Edge R., Truscott T.G. (2023). Photochemical and Photophysical Properties of Carotenoids and Reactive Oxygen Species: Contradictions Relating to Skin and Vision. Oxygen.

[90] Chisté R.C., Freitas M., Mercadante A.Z., Fernandes E. (2014). Carotenoids inhibit lipid peroxidation and hemoglobin oxidation, but not the depletion of glutathione induced by ROS in human erythrocytes. Life Sci..

[91] Jafari Z., Bigham A., Sadeghi S., Dehdashti S.M., Rabiee N., Abedivash A., Bagherzadeh M., Nasseri B., Karimi-Maleh H., Sharifi E. (2022). Nanotechnology-Abetted Astaxanthin Formulations in Multimodel Therapeutic and Biomedical Applications. J. Med. Chem..

[92] Juan C.A., Pérez de la Lastra J.M., Plou F.J., Pérez-Lebeña E. (2021). The Chemistry of Reactive Oxygen Species (ROS) Revisited: Outlining Their Role in Biological Macromolecules (DNA, Lipids and Proteins) and Induced Pathologies. Int. J. Mol. Sci..

[93] Chaudhary P., Janmeda P., Docea A.O., Yeskaliyeva B., Razis A.F.A., Modu B., Calina D., Sharifi-Rad J. (2023). Oxidative stress, free radicals and antioxidants: Potential crosstalk in the pathophysiology of human diseases. Front. Chem..

[94] Genç Y., Bardakci H., Yücel Ç., Karatoprak G.Ş., Küpeli Akkol E., Hakan Barak T., Sobarzo-Sánchez E. (2020). Oxidative Stress and Marine Carotenoids: Application by Using Nanoformulations. Mar. Drugs.

[95] Salminen A., Kaarniranta K., Kauppine A. (2022). Photoaging: UV radiation-induced inflammation and immunosuppression accelerate the aging process in the skin. Inflamm. Res..

[96] Catanzaro E., Bishayee A., Fimognari C. (2020). On a Beam of Light: Photoprotective Activities of the Marine Carotenoids Astaxanthin and Fucoxanthin in Suppression of Inflammation and Cancer. Mar. Drugs.

[97] Balić A., Mokos M. (2019). Do We Utilize Our Knowledge of the Skin Protective Effects of Carotenoids Enough?. Antioxidants.

[98] Anbualakan K., Tajul Urus N.Q., Makpol S., Jamil A., Mohd Ramli E.S., Md Pauzi S.H., Muhammad N. (2023). A Scoping Review on the Effects of Carotenoids and Flavonoids on Skin Damage Due to Ultraviolet Radiation. Nutrients.

[99] Darvin M.E., Lademann J., von Hagen J., Lohan S.B., Kolmar H., Meinke M.C., Jung S. (2022). Carotenoids in Human Skin In Vivo: Antioxidant and Photo-Protectant Role against External and Internal Stressors. Antioxidants.

[100] Bono M.R., Tejon G., Flores-Santibañez F., Fernandez D., Rosemblatt M., Sauma D. (2016). Retinoic Acid as a Modulator of T Cell Immunity. Nutrients.

[101] Oliveira L.M., Teixeira F.M.E., Sato M.N. (2018). Impact of Retinoic Acid on Immune Cells and Inflammatory Diseases. Mediat. Inflamm..

[102] Wynn T.A., Vannella K.M. (2016). Macrophages in Tissue Repair, Regeneration, and Fibrosis. Immunity.

[103] Brancewicz J., Wójcik N., Sarnowska Z., Robak J., Król M. (2025). The Multifaceted Role of Macrophages in Biology and Diseases. Int. J. Mol. Sci..

[104] Wang C., Ma C., Gong L., Guo Y., Fu K., Zhang Y., Zhou H., Li Y. (2021). Macrophage Polarization and Its Role in Liver Disease. Front. Immunol..

[105] Lis-López L., Bauset C., Seco-Cervera M., Cosín-Roger J. (2021). Is the Macrophage Phenotype Determinant for Fibrosis Development?. Biomedicines.

[106] Chang M.X., Xiong F. (2020). Astaxanthin and its Effects in Inflammatory Responses and Inflammation-Associated Diseases: Recent Advances and Future Directions. Molecules.

[107] Pružinská K., Chrastina M., Khademnematolahi S., Vyletelová V., Gajdošová L., Pastvová L., Dráfi F., Poništ S., Pašková Ľ., Kucharská J. (2024). Astaxanthin, Compared to Other Carotenoids, Increases the Efficacy of Methotrexate in Rat Adjuvant Arthritis. Int. J. Mol. Sci..

[108] Ávila-Román J., García-Gil S., Rodríguez-Luna A., Motilva V., Talero E. (2021). Anti-Inflammatory and Anticancer Effects of Microalgal Carotenoids. Mar. Drugs.

[109] Baeza-Morales A., Medina-García M., Martínez-Peinado P., Pascual-García S., Pujalte-Satorre C., López-Jaén A.B., Martínez-Espinosa R.M., Sempere-Ortells J.M. (2024). The Antitumour Mechanisms of Carotenoids: A Comprehensive Review. Antioxidants.

[110] Joshi M., Hiremath P., John J., Ranadive N., Nandakumar K., Mudgal J. (2023). Modulatory role of vitamins A, B3, C, D, and E on skin health, immunity, microbiome, and diseases. Pharmacol. Rep..

[111] Camera E., Mastrofrancesco A., Fabbri C., Daubrawa F., Picardo M., Sies H., Stahl W. (2009). Astaxanthin, canthaxanthin and beta-carotene differently affect UVA-induced oxidative damage and expression of oxidative stress-responsive enzymes. Exp. Dermatol..

[112] Park S. (2022). Biochemical, structural and physical changes in aging human skin, and their relationship. Biogerontology.

[113] Jadach B., Mielcarek Z., Osmałek T. (2024). Use of Collagen in Cosmetic Products. Curr. Issues Mol. Biol..

[114] Shoulders M.D., Raines R.T. (2009). Collagen structure and stability. Annu. Rev. Biochem..

[115] Boraldi F., Lofaro F.D., Bonacorsi S., Mazzilli A., Garcia-Fernandez M., Quaglino D. (2024). The Role of Fibroblasts in Skin Homeostasis and Repair. Biomedicines.

[116] Papakonstantinou E., Roth M., Karakiulakis G. (2012). Hyaluronic acid: A key molecule in skin aging. Dermato-Endocrinology.

[117] Iaconisi G.N., Lunetti P., Gallo N., Cappello A.R., Fiermonte G., Dolce V., Capobianco L. (2023). Hyaluronic Acid: A Powerful Biomolecule with Wide-Ranging Applications—A Comprehensive Review. Int. J. Mol. Sci..

[118] Lierova A., Kasparova J., Filipova A., Cizkova J., Pekarova L., Korecka L., Mannova N., Bilkova Z., Sinkorova Z. (2022). Hyaluronic Acid: Known for Almost a Century, but Still in Vogue. Pharmaceutics.

[119] Crupi P., Faienza M.F., Naeem M.Y., Corbo F., Clodoveo M.L., Muraglia M. (2023). Overview of the Potential Beneficial Effects of Carotenoids on Consumer Health and Well-Being. Antioxidants.

[120] Li Y., Zhao Y., Zhang H., Ding Z., Han J. (2024). The Application of Natural Carotenoids in Multiple Fields and Their Encapsulation Technology: A Review. Molecules.

[121] Fluhr J.W., Muguet V., Christen-Zaech S. (2025). Restoring Skin Hydration and Barrier Function: Mechanistic Insights Into Basic Emollients for Xerosis Cutis. Int. J. Dermatol..

[122] Fluhr J.W., Alexis A.F., Andriessen A., Barrios O.L.F., Bjerring P., Foley P., Gold M.H., Kaderbhai H., Zhang C. (2024). A global perspective on the treatment and maintenance of mature skin using gentle cleansers and moisturizers. Int. J. Dermatol..

[123] Załęcki P., Rogowska K., Wąs P., Łuczak K., Wysocka M., Nowicka D. (2024). Impact of Lifestyle on Differences in Skin Hydration of Selected Body Areas in Young Women. Cosmetics.

[124] Bakac E.R., Percin E., Gunes-Bayir A., Dadak A. (2023). A Narrative Review: The Effect and Importance of Carotenoids on Aging and Aging-Related Diseases. Int. J. Mol. Sci..

[125] Bollag W.B., Aitkens L., White J., Hyndman K.A. (2020). Aquaporin-3 in the epidermis: More than skin deep. Am. J. Physiol.-Cell Physiol..

[126] Qin H., Zheng X., Zhong X., Shetty A.K., Elias P.M., Bollag W.B. (2011). Aquaporin-3 in keratinocytes and skin: Its role and interaction with phospholipase D2. Arch Biochem. Biophys..

[127] Ren Q., Qu L., Yuan Y., Wang F. (2024). Natural Modulators of Key Signaling Pathways in Skin Inflammageing. Clin. Cosmet. Investig. Dermatol..

[128] Donato A., Belluzzi E., Mattiuzzo E., Venerando R., Cadamuro M., Ruggieri P., Vindigni V., Brun P. (2022). Anti-Inflammatory and Pro-Regenerative Effects of Hyaluronan-Chitlac Mixture in Human Dermal Fibroblasts: A Skin Ageing Perspective. Polymers.

[129] Hänel K.H., Cornelissen C., Lüscher B., Baron J.M. (2013). Cytokines and the Skin Barrier. Int. J. Mol. Sci..

[130] Singampalli K.L., Balaji S., Wang X., Parikh U.M., Kaul A., Gilley J., Birla R.K., Bollyky P.L., Keswani S.G. (2020). The Role of an IL-10/Hyaluronan Axis in Dermal Wound Healing. Front. Cell Dev. Biol..

[131] Johnson K.E., Wilgus T.A. (2014). Vascular Endothelial Growth Factor and Angiogenesis in the Regulation of Cutaneous Wound Repair. Adv. Wound Care.

[132] Gibbs S., Silva Pinto A.N., Murli S., Huber M., Hohl D., Ponec M. (2000). Epidermal growth factor and keratinocyte growth factor differentially regulate epidermal migration, growth, and differentiation. Wound Repair Regen..

[133] Gupta A., Singh A.P., Singh V.K., Singh P.R., Jaiswal J., Kumari N., Upadhye V., Singh S.C., Sinha R.P. (2023). Natural Sun-Screening Compounds and DNA-Repair Enzymes: Photoprotection and Photoaging. Catalysts.

[134] Michalak M., Pierzak M., Kręcisz B., Suliga E. (2021). Bioactive Compounds for Skin Health: A Review. Nutrients.

[135] Lin T.-K., Zhong L., Santiago J.L. (2018). Anti-Inflammatory and Skin Barrier Repair Effects of Topical Application of Some Plant Oils. Int. J. Mol. Sci..

[136] Wu H.J., Wu E. (2012). The role of gut microbiota in immune homeostasis and autoimmunity. Gut Microbes.

[137] Jimenez-Sanchez M., Celiberto L.S., Yang H., Sham H.P., Vallance B.A. (2025). The gut-skin axis: A bi-directional, microbiota-driven relationship with therapeutic potential. Gut Microbes.

[138] Ma Z.F., Lee Y.Y. (2025). The Role of the Gut Microbiota in Health, Diet, and Disease with a Focus on Obesity. Foods.

[139] Salem I., Ramser A., Isham N., Ghannoum M.A. (2018). The Gut Microbiome as a Major Regulator of the Gut-Skin Axis. Front. Microbiol..

[140] Jiang Z., Mei L., Li Y., Guo Y., Yang B., Huang Z., Li Y. (2024). Enzymatic Regulation of the Gut Microbiota: Mechanisms and Implications for Host Health. Biomolecules.

[141] Boev M., Stănescu C., Turturică M., Cotârleţ M., Batîr-Marin D., Maftei N., Chiţescu C., Grigore-Gurgu L., Barbu V., Enachi E. (2024). Bioactive Potential of Carrot-Based Products Enriched with Lactobacillus plantarum. Molecules.

[142] Reboul E. (2019). Mechanisms of Carotenoid Intestinal Absorption: Where Do We Stand?. Nutrients.

[143] Widjaja-Adhi M.A.K., Golczak M. (2020). The molecular aspects of absorption and metabolism of carotenoids and retinoids in vertebrates. Biochim. Biophys. Acta (BBA)-Mol. Cell Biol. Lipids.

[144] Reboul E. (2013). Absorption of vitamin A and carotenoids by the enterocyte: Focus on transport proteins. Nutrients.

[145] Eroglu A., Al’Abri I.S., Kopec R.E., Crook N., Bohn T. (2023). Carotenoids and Their Health Benefits as Derived via Their Interactions with Gut Microbiota. Adv. Nutr..

[146] Randeni N., Bordiga M., Xu B. (2024). A Comprehensive Review of the Triangular Relationship among Diet–Gut Microbiota–Inflammation. Int. J. Mol. Sci..

[147] Munteanu C., Schwartz B. (2024). Interactions between Dietary Antioxidants, Dietary Fiber and the Gut Microbiome: Their Putative Role in Inflammation and Cancer. Int. J. Mol. Sci..

[148] Krishnamurthy H.K., Pereira M., Bosco J., George J., Jayaraman V., Krishna K., Wang T., Bei K., Rajasekaran J.J. (2023). Gut commensals and their metabolites in health and disease. Front. Microbiol..

[149] Abrante-Pascual S., Nieva-Echevarría B., Goicoechea-Oses E. (2024). Vegetable Oils and Their Use for Frying: A Review of Their Compositional Differences and Degradation. Foods.

[150] Schwingshackl L., Bogensberger B., Benčič A., Knüppel S., Boeing H., Hoffmann G. (2018). Effects of oils and solid fats on blood lipids: A systematic review and network meta-analysis. J. Lipid Res..

[151] Verge-Mèrida G., Barroeta A.C., Ferrer C., Serrano T., Guardiola F., Soler M.D., Sala R. (2022). Olive Pomace and Soybean-Sunflower Acid Oils as Alternative Fat Sources in European Seabass (*Dicentrarchus labrax*) Diets: Effects on Performance, Digestibility and Flesh Fatty Acid Composition and Quality Parameters. Animals.

[152] Rosqvist F., Niinistö S. (2024). Fats and oils—A scoping review for Nordic Nutrition Recommendations 2023. Food Nutr. Res..

[153] Spiteller G. (2005). The relation of lipid peroxidation processes with atherogenesis: A new theory on atherogenesis. Mol. Nutr. Food Res..

[154] Greenberg M.E., Li X.M., Gugiu B.G., Gu X., Qin J., Salomon R.G., Hazen S.L. (2008). The lipid whisker model of the structure of oxidized cell membranes. J. Biol. Chem..

[155] Duché G., Sanderson J.M. (2024). The Chemical Reactivity of Membrane Lipids. Chem. Rev..

[156] Tan B.L., Norhaizan M.E., Liew W.P., Sulaiman Rahman H. (2018). Antioxidant and Oxidative Stress: A Mutual Interplay in Age-Related Diseases. Front. Pharmacol..

[157] Valgimigli L. (2023). Lipid Peroxidation and Antioxidant Protection. Biomolecules.

[158] Kilicarslan You D., Fuwad A., Lee K.H., Kim H.K., Kang L., Kim S.M., Jeon T.-J. (2024). Evaluation of the Protective Role of Vitamin E against ROS-Driven Lipid Oxidation in Model Cell Membranes. Antioxidants.

[159] Salehi B., Azzini E., Zucca P., Maria Varoni E., Anil Kumar N.V., Dini L., Panzarini E., Rajkovic J., Valere Tsouh Fokou P., Peluso I. (2020). Plant-Derived Bioactives and Oxidative Stress-Related Disorders: A Key Trend towards Healthy Aging and Longevity Promotion. Appl. Sci..

[160] Athanasopoulou S., Spanidi E., Panagiotidou E., Cavagnino A., Bobier A., Gardikis K. (2024). An Advanced Combinatorial System from *Vitis vinifera* Leaves and Propolis Enhances Antioxidants’ Skin Delivery and Fibroblasts Functionality. Pharmaceuticals.

[161] Gammone M.A., Riccioni G., D'Orazio N. (2015). Marine Carotenoids against Oxidative Stress: Effects on Human Health. Mar. Drugs..

[162] Sumalla-Cano S., Eguren-García I., Lasarte-García Á., Prola T.A., Martínez-Díaz R., Elío I. (2024). Carotenoids Intake and Cardiovascular Prevention: A Systematic Review. Nutrients.

[163] Tamas C., Hreniuc I.M.J., Tecuceanu A., Ciuntu B.M., Ibanescu C.L., Tamas I., Ianole V., Stanescu C., Pintilie C.T., Zamfir C.L. (2021). Non-Melanoma Facial Skin Tumors—The Correspondence between Clinical and Histological Diagnosis. Appl. Sci..

[164] Anghel L., Boev M., Stanescu C., Mitincu Caramfil S., Luca L., Muşat C.L., Ciubara A. (2023). Depression in the diabetic patient. BRAIN Broad Res. Artif. Intell. Neurosci..

[165] Batir-Marin D., Ștefan C.S., Boev M., Gurău G., Popa G.V., Matei M.N., Ursu M., Nechita A., Maftei N.-M. (2025). A Multidisciplinary Approach of Type 1 Diabetes: The Intersection of Technology, Immunotherapy, and Personalized Medicine. J. Clin. Med..

[166] Ohrnberger J., Sutton M., Fichera E. (2016). The dynamics of physical and mental health in the older population. J. Econ. Ageing.

[167] Hernandez R., Boughton S.W., Schuette S.A., Moskowitz J.T., Bassett S.M., Shiu E.W. (2017). Psychological Well-being and Physical Health: Associations, Mechanisms, and Future Directions. Emot. Rev..

[168] Stanescu C., Anghel L., Tamas C., Ciubara A. (2025). Education of Patients and Their Families to Manage Emotional Impact of Skin Scars. BRAIN Broad Res. Artif. Intell. Neurosci..

[169] Lem K., McGilton K.S., Aelick K., Iaboni A., Babineau J., Colborne D.H., Edwards C., Bretzlaff M., Lender D., Gibson J.-L. (2021). Social connection and physical health outcomes among long-term care home residents: A scoping review. BMC Geriatr..

[170] Stanescu C., Boev M., Avram O.A., Ciubara A. (2024). Investigating the Connection Between Skin Cancers and Mental Disorders: A Thorough Analysis. BRAIN Broad Res. Artif. Intell. Neurosci..

[171] Anghel L., Ciubară A., Nechita A., Nechita L., Manole C., Baroiu L., Ciubară A.B., Mușat C.L. (2023). Sleep Disorders Associated with Neurodegenerative Diseases. Diagnostics.

[172] Knaggs H., Lephart E.D. (2023). Enhancing Skin Anti-Aging through Healthy Lifestyle Factors. Cosmetics.

[173] Li X., Wang S. (2025). Dose-response relationship between carotenoid intake and risk of depressive symptoms in postmenopausal women. Front. Psychiatry.

[174] Shin J., Song M.-H., Oh J.-W., Keum Y.-S., Saini R.K. (2020). Pro-oxidant Actions of Carotenoids in Triggering Apoptosis of Cancer Cells: A Review of Emerging Evidence. Antioxidants.

[175] Eghbaliferiz S., Iranshahi M. (2016). Prooxidant Activity of Polyphenols, Flavonoids, Anthocyanins and Carotenoids: Updated Review of Mechanisms and Catalyzing Metals. Phytother. Res..

[176] Nishino H., Masuda M., Takayasu J., Murakoshi M., Yano M., Tsuruta J., Tsuruta J., Okuda M., Khachik F. (2000). Cancer prevention by natural carotenoids. BioFactors..

